# Ovarian insufficiency and CTNNB1 mutations drive malignant transformation of endometrial hyperplasia with altered PTEN/PI3K activities

**DOI:** 10.1073/pnas.1814506116

**Published:** 2019-02-19

**Authors:** Jumpei Terakawa, Vanida Ann Serna, Makoto Mark Taketo, Takiko Daikoku, Adrian A. Suarez, Takeshi Kurita

**Affiliations:** ^a^Department of Cancer Biology and Genetics, Ohio State University, Columbus, OH 43210;; ^b^The Comprehensive Cancer Center, Ohio State University, Columbus, OH 43210;; ^c^Division of Experimental Therapeutics, Graduate School of Medicine, Kyoto University, Yoshida-Konoe-cho, Sakyo-ku, 606-8506 Kyoto, Japan;; ^d^Division of Transgenic Animal Science, Advanced Science Research Center, Kanazawa University, 920-8640 Kanazawa, Japan;; ^e^Department of Pathology, Ohio State University, Columbus, OH 43210

**Keywords:** metastasis, menopause, estrogen, progesterone, myometrial invasion

## Abstract

Endometrioid endometrial carcinomas (EECs) carry multiple driver mutations even when they are low grade. However, the biological significance of these concurrent mutations is unknown. We explored the interactions among three signature EEC mutations: loss-of-function (LOF) mutations in *PTEN*, gain-of-function (GOF) mutations of phosphoinositide 3-kinase (PI3K), and *CTNNB1* exon 3 mutations, utilizing in vivo mutagenesis of the mouse uterine epithelium. While epithelial cells with a monoallelic mutation in any one of three genes failed to propagate in the endometrium, any combination of two or more mutant alleles promoted the growth of epithelium, causing simple hyperplasia, in a dose-dependent manner. Notably, *Ctnnb1* exon 3 deletion significantly increased the size of hyperplastic lesions by promoting the growth of PTEN LOF and/or PI3K GOF mutant cells through the activation of neoadenogenesis pathways. Although these three mutations were insufficient to cause EEC in intact female mice, castration triggered malignant transformation, leading to myometrial invasion and serosal metastasis. Treatment of castrated mice with progesterone or estradiol attenuated the neoplastic transformation. This study demonstrates that multiple driver mutations are required for premalignant cells to break the growth-repressing field effect of normal endometrium maintained by ovarian steroids and that *CTNNB1* exon 3 mutations play critical roles in the growth of preneoplastic cells within the endometrium of premenopausal women and in the myometrial invasion of EECs in menopausal women.

## Significance

Our mouse model of endometrial cancer (EC) reveals the mechanism underlying the presence of multiple driver mutations in early EC along with the menopause-associated risk in endometrial carcinogenesis. Human ECs carry multiple driver mutations even when they are low grade, whereas a single driver mutation caused EC in previous mouse studies. Our study established that multiple driver mutations are essential for clonal expansion of preneoplastic epithelial cells within the anticancer environment of the normal endometrium. The tumor-suppressing activity of normal endometrium is maintained by estradiol and progesterone, indicating that menopause itself can promote ECs. In the absence of estradiol and progesterone, a *CTNNB1* mutation drives myometrial invasion and metastasis of ECs.

Endometrial carcinoma (EC) is the most common gynecologic malignancy in the United States and accounts for 7% of all cancers in women (1). Recent large-scale comprehensive genomic analysis by The Cancer Genome Atlas (TCGA) proposed the classification of EC subtypes by unique molecular features: *POLE* ultramutated, microsatellite instability (MSI) hypermutated, copy-number-low (CL; endometrioid), and copy-number-high (CH; serous-like) EC subtypes (2). This molecular classification demonstrates clear molecular features associated with certain histopathological subtypes.

Among all ECs, CL EC is the most common subtype by molecular classification. In TCGA dataset, 98% (88/90 cases) of the CL EC cases had endometrioid EC (EEC) histopathology (CL-EEC). In the same dataset, *PTEN* (phosphatase and tensin homolog deleted on chromosome 10) mutations were detected in 77% (68/88) of CL-EECs, whereas *PTEN* mutations were present only in 15% (9/60) of CH-ECs (2–4). In CL-EECs, a gain of function (GOF) mutation in the p110α catalytic subunit (*PIK3CA*) and a loss-of-function (LOF) mutation in the p85α regulatory subunit (*PIK3R1*) of phosphoinositide 3-kinase (PI3K) are mutually exclusive. These mutations were found in 81% (71/88) of CL-EECs. Their mutual exclusivity is explained by the functional redundancy of these mutations, as they result in the constitutive activation of PI3K (5). However, *PTEN* and PI3K (*PIK3CA* or *PIK3R1*) mutations co-occurred in 67% (59/88) of CL-EECs. This observation questions the widely accepted concept that the loss of PTEN and activation of PI3K have synonymous effects on cellular physiology, as they catalyze opposite reactions: PI3K converts phosphatidylinositol (4,5)-biphosphate (PIP2) to phosphatidylinositol (3,4,5)-trisphosphate (PIP3), whereas PTEN converts PIP3 to PIP2.

In general, EECs carry multiple driver mutations with well-characterized carcinogenic potential. In TCGA EC dataset, missense mutations of *CTNNB1* in exon 3 were present in 53% (47/88) of CL-EEC cases, making them the third most frequent mutation following *PTEN* and PI3K. Missense mutations to D32, D33, G34, S37, T41, and S45, all of which are encoded by exon 3, stabilize β-catenin/CTNNB1 protein by removing the target of the destruction complex and, thus, activate the transcriptional targets of CTNNB1-TCF (T cell factor)/LEF (lymphoid enhancer-binding factor) (6). Because two-thirds (32/47) of the *CTNNB1* mutant EECs carry mutations in both *PTEN* and *PI3KCA*/*PIK3R1*, these three mutations likely have synergistic or additive effects in EEC pathogenesis. The high prevalence of these three gene defects in low-grade ECs and the absence of association with grade, stage, and clinical outcomes strongly suggest that they play a critical role in the initiation and early progression of EECs (7). In fact, a recent study demonstrated that *CTNNB1* mutations are associated with a 5.97 hazard ratio (95% CI 2.69–13.21) for recurrence when the analysis was limited to early stage EECs [The International Federation of Gynecology and Obstetrics (FIGO) grade 1 or 2 and stage I or II] (8). Thus, in this current study, we explored the combinatorial effects of PTEN LOF, PI3K GOF, and *CTNNB1* exon 3 mutations (CTNNB1 GOF) on early endometrial carcinogenesis utilizing genetically engineered mouse models.

Genetically engineered mouse models help establish the oncogenic potential of mutations that recur in human ECs (9, 10). However, there are limitations to mouse EC models when utilizing Cre-transgenic lines, a standard approach for modeling cancers in mice. For example, commonly used Cre-transgenic lines express Cre in the embryonic and neonatal uterus before the epithelial cells establish their uterine identity (11). In addition, because of the widespread Cre expression in the uterine epithelium (UtE), ECs can develop without clonal expansion of mutant cells, an essential step for carcinogenesis (12). Accordingly, mouse EC models utilizing Cre transgenes are not suitable to study the combinational function of PTEN, PI3K, and CTNNB1 mutations in early endometrial carcinogenesis and clonal expansion. Hence, we induced mutations in a small subset of differentiated uterine epithelial cells in mice by adenovirus-Cre (Ad-Cre). Utilizing this mouse model, we studied the collaborative effects of the three most prevalent mutations in human EECs on the initiation and progression of EECs. We also investigated the effects of ovarian insufficiency in endometrial carcinogenesis. EECs are commonly referred to as type I ECs, for which an increase in estrogen exposure is a known risk factor (13). EECs typically occur in postmenopausal women who have low systemic estrogen levels. This paradoxical correlation between low estrogenic activity and EEC incidence may be explained by other menopause-associated factors, including age and loss of menstruation. Given that these factors can be excluded in the mouse model, we explored the direct effect of ovarian insufficiency on endometrial carcinogenesis.

## Results

### Combination Effects of Mutant Alleles in Endometrial Pathogenesis.

**Fig. 1.**
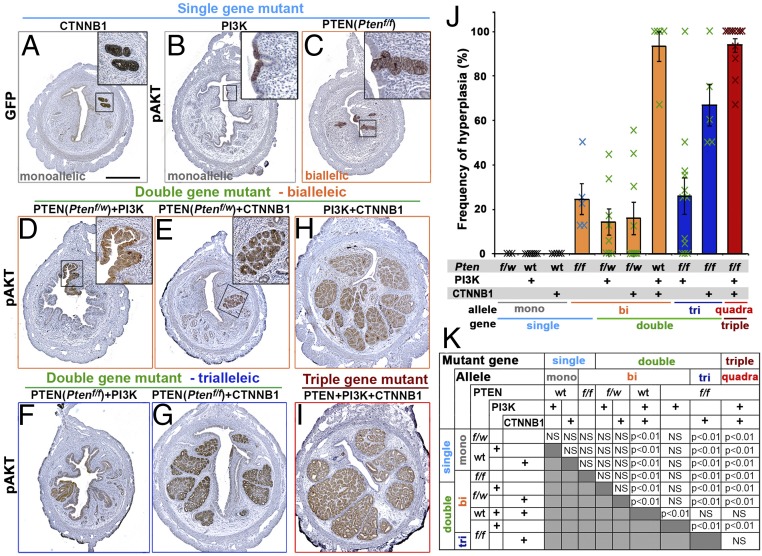
Genotype-dependent uterine histopathology at 2 mo after in vivo mutagenesis. Immunohistochemical analyses for green fluorescent protein (GFP) (markers for Cre activity) (*A*) and pAKT (*B*–*I*) highlighted the mutant epithelial cells in the uteri from mice carrying indicated mutation(s). (Scale bar: 500 μm.) (*J* and *K*) Frequency of hyperplastic lesions in mice with different genotypes. (*J*) Result is presented by average ± SE of all mice in each group including ones negative for epithelial lesions. (*K*) Summary of statistical analysis by one-way ANOVA with post hoc Tukey’s HSD test.

PTEN LOF, PI3K-activating, and CTNNB1 exon 3 mutations were induced in a small subset of uterine epithelial cells by the infusion of Ad-Cre into the uterine cavity of mice carrying *Pten* floxed (*Pten*^*f*^) (14), a constitutively active mutant PI3K knock-in (*ROSA*^Pik3ca^^*^) (15), and *Ctnnb1* exon 3 floxed (*Ctnnb1*^*f(ex3)*^) (16) alleles (*SI Appendix*, Fig. S1). Cre activity was tracked by the expression of membrane-targeted enhanced green fluorescent protein (mEGFP) from *ROSA*^*TE*^ allele (17). To evaluate the direct effects of mutations on endometrial phenotypes, the histopathology of uteri was compared between different genotypes at 2 mo after mutagenesis (Fig. 1). By 2 mo, mutant cells have had sufficient time to grow, but additional alterations are unlikely to have occurred. Since there was no gross tumor detected within 2 mo (*SI Appendix*, Fig. S1*G*), the dosage effect of mutant alleles was histologically assessed as summarized in Fig. 1 and Table 1.

**Table 1.** Summary: Genotype and uterine pathology in intact mice

Genotype					Postinjection months/Frequency of hyperplasia, %								
					2 mo			3 mo/4 mo^†^			6 mo		
Mutant gene	Allele	*Pten*	PI3K	CTNNB1	*n*	Per mouse	Per section	*n*	Per mouse	Per section	*n*	Per mouse	Per section
Single	mono	*f/w*	—	wt	3	0 (0/3)	0	—	—	—	—	—	—
		wt	Pik3ca*	wt	7	0 (0/7)	0	8^‡^	0 (0/8)	0	—	—	—
		wt	—	*f(ex3)/w*	6	0 (0/6)	0	4	0 (0/4)	0	3	0 (0/3)	0
	bi	*f/f*	—	wt	5	100 (5/5)	24.4 ± 6.9	9	100 (9/9)	40.1 ± 6.9	5	100 (5/5)	73.4 ± 8.3
Double	bi	*f/w*	Pik3ca*	wt	8	62.5 (5/8)	14.1 ± 5.9	—	—	—	—	—	—
		*f/w*	—	*f(ex3)/w*	9	44.4 (4/9)	15.9 ± 7.4	—	—	—	—	—	—
		wt	Pik3ca*	*f(ex3)/w*	5	100 (5/5)	93.3 ± 6.7	3^†^	100 (3/3)	100.0 ± 0.0	—	—	—
	tri	*f/f*	Pik3ca*	wt	12	75 (9/12)	25.9 ± 8.3	4^†^	100 (4/4)	81.9 ± 11.9	—	—	—
		*f/f*	—	*f(ex3)/w*	5	100 (5/5)	67.0 ± 9.4	7	100 (7/7)	82.1 ± 8.7	6	100 (6/6)	89.1 ± 5.6^§^
Triple	quadra^¶^	*f/f*	Pik3ca*	*f(ex3)/w*	12	100 (12/12)	93.8 ± 3.2	3^†^	100 (3/3)	100.0 ± 0.0	—	—	—

Frequency of hyperplasia per section is the percentage of uterine sections that contain hyperplasia in each mouse. It is expressed as mean ± SE among all mice in each group. F, floxed allele; w, wild-type allele; wt, wild type.

^†^ *n* = 3 mo.

^‡^ *n* = 4 each for 3 and 4 mo.

^§^ Endometrioid endometrial carcinoma was detected in 40% of uterine sections in one mouse out of six (16.7%).

^¶^ Quadra-allelic mutant cells (PIK3CA*-positive, FOXA2/RUNX2-positive, and PTEN-null) were not detected.

Epithelial cells with a monoalleic mutation in one of three genes failed to develop histologically recognizable lesions within 2 mo. When tracked by mEGFP reporter for Cre activity, epithelial cells with CTNNB1 exon 3 deletion mutation were always found in uterine glands (Fig. 1*A*). Although the expression of PIK3CA* from the *ROSA*^Pik3ca*^ allele increased phospho-AKT Ser473 (pAKT), the mutant cells remained dormant, forming small patches within the luminal epithelium (Fig. 1*B*). There are multiple reports that *Pten* haploinsufficiency results in abnormal endometrial epithelial proliferation (10, 18). However, there was no detectable effect of monoallelic loss of *Pten* (*Pten*^*f/w*^) alone on the UtE in histological analyses, including pAKT immunohistochemistry, at 2 mo (*SI Appendix*, Fig. S2*A*).

In contrast to monoalleleic mutations, combinations of two mutant alleles induced epithelial lesions, demonstrating synergy. Biallelic loss of *Pten* (*Pten*^*f/f*^) consistently resulted in hyperplasia with elevated pAKT in 2 mo (Fig. 1*C* and *SI Appendix*, Fig. S2*B*). *Pten*^*f/w*^ in combination with CTNNB1 mutation stimulated the growth of epithelial cells with up-regulation of pAKT (Fig. 1*E*). Synergy between PTEN and CTNNB1 mutations was demonstrated by the development of glandular hyperplasia with *Pten*^*f/w*^ and CTNNB1 mutation (Fig. 1*E*), each of which alone did not induce epithelial growth. There was a strong *Pten* gene-dose dependency in CTNNB1 mutant cells (Table 1, *P* < 0.01): When CTNNB1 mutation was combined with *Pten*^*f/w*^, hyperplasia was detected in only 44% (4/9) of mice (Fig. 1*E*) with a frequency (hyperplasia positive sections/total sections) of 15.9 ± 7.4% (average ± SE), whereas the combination of CTNNB1 mutation with *Pten*^*f/f*^ resulted in large glandular hyperplasia in 100% (5/5) of mice with a frequency of 67.0 ± 9.4% (Fig. 1*G* and Table 1).

Growth of PI3K mutant cells was also promoted by monoallelic loss of *Pten* (Fig. 1*D*). However, there was no evidence for a *Pten* gene-dose effect, as the phenotypes and frequency of the PI3K mutant lesions were not significantly different between mice carrying *Pten*^*f/w*^ and *Pten*^*f/f*^ (Fig. 1 *D* and *F* and Table 1). Immunostaining revealed that hyperplastic lesions in *Pten*^*f/f*^*; ROSA*^Pik3ca*^ mice were positive for PIK3CA* as well as PTEN (*SI Appendix*, Fig. S2*C*). However, PTEN staining was weaker in these epithelial lesions, indicating that these cells lost one allele of *Pten* (*SI Appendix*, Fig. S2 *C*, *b*). Meanwhile, PTEN-null lesions were rare and found only in 25% of *Pten*^*f/f*^*; ROSA*^Pik3ca*^ mice (3/12). These lesions were always negative for PIK3CA*, suggesting either Ad-Cre was insufficient to recombine three alleles simultaneously (two *Pten* floxed alleles + one *ROSA*^Pik3ca*^ allele) or the cells carrying these three mutant alleles did not grow. Then, we analyzed *Pten*^*f/f*^*; ROSA*^Pik3ca*^ mice just 7 d after mutagenesis and found a small number of cells carrying PTEN-null and PI3K mutations in all mice (*n* = 4) (*SI Appendix*, Fig. S2 *C*, *d*–*i*). This observation would be consistent with an eventual loss of triallelic-mutant cells (PTEN-null and PIK3CA* positive), potentially due to an innate tumor-suppressing mechanism that blocks the propagation of cells containing excessive PIP3 by arresting growth and/or promoting apoptosis (19–22). Interestingly, PTEN-null/PIK3CA*-positive cells were often found in the apical layer of epithelium. Thus, these cells may be removed from the epithelium by neighboring normal cells through apical exclusion (23).

A synergistic effect was also prominent between PI3K and CTNNB1 mutations, which resulted in large glandular hyperplasia lesions similar to *Pten*^*f/f*^ and CTNNB1 double-gene/triallelic (as referred to PTEN+CTNNB1 hereafter) mutations (Fig. 1*H*). When three gene mutations were combined (*Pten*^*f/f*^; *ROSA*^Pik3ca*^; *Ctnnb1*^*f(ex3)*^), triple-gene (quadra-allelic) mutant mice also developed glandular hyperplasia in the uterus (Fig. 1*I*). However, the combinatorial effect of three mutant genes was not quantitatively detected by the histopathological analysis (Fig. 1 *J* and *K*), and there was no statistical difference in the frequency of hyperplasia between PTEN+CTNNB1, PI3K+CTNNB1, and triple-gene mutant mice (Fig. 1*K*).

### Progression of Epithelial Lesions with PTEN and PI3K Mutations.

**Fig. 2.**
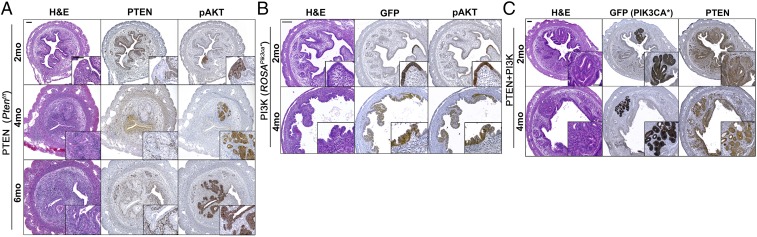
Histopathological analyses of the uteri with PTEN LOF and/or PI3K GOF mutation. Biallelic loss of *Pten* (*A*), GOF mutation of PI3K (*B*), and the combination of loss of *Pten* and PI3K GOF (*C*) followed up to 6 mo. *Insets* (IHC): Epithelial lesions were highlighted by loss of PTEN proteins (*A*), Cre activation (GFP) (*B* and *C*), and phosphorylation of AKT (*A*–*C*). (Scale bars: 100 μm.)

When mice were followed up to 6 mo, PTEN-null lesions gradually expanded within the endometrium, and the frequency of hyperplasia was significantly higher at 6 mo compared with 2 mo (*P* < 0.01) and 4 mo (*P* < 0.05) (Fig. 2*A* and Table 1). However, biallelic loss of *Pten* never induced EC. Loss of PTEN always coincided with elevated pAKT in the epithelium (Fig. 2*A* and *SI Appendix*, Fig. S2*B*). In addition, PTEN-null hyperplastic lesions were associated with stromal cells that had elevated PTEN expression (*SI Appendix*, Fig. S2*B*, arrow), indicating interactions between epithelial and stromal tissues.

In mice carrying the *ROSA*^Pik3ca*^ allele, uterine tissue architecture was distorted by 3 mo, due to pressure in the uterovaginal canal caused by vulva tumors (*SI Appendix*, Fig. S1*E*). As the accuracy of histopathological analysis was compromised, focal epithelial stratification was excluded from the category of pathological phenotypes. Because of the vulvar tumors, *ROSA*^Pik3ca*^ mice were euthanized by 4 mo after Ad-Cre injection. Although PI3K mutant cells gradually expanded from 2 to 4 mo (Fig. 2*B*), morphologically identifiable hyperplastic lesions did not develop within this timeframe.

We pursued the combinatorial effect of PTEN and PI3K mutations further in *Pten*^*f/f*^*; ROSA*^Pik3ca*^ mice. However, PTEN-null and PIK3CA* expression was always exclusive. Monoallelic *Pten* loss in combination with PI3K activating mutation promoted epithelial growth into the endometrial stroma (Fig. 2*C*). Nevertheless, growth was restricted to small stromal areas at 4 mo, and cellular morphology was hypertrophic but essentially normal (Fig. 2*C*).

### CTNNB1 Mutation in Uterine Epithelial Cells Initiates Uterine Adenogenesis.

**Fig. 3.**
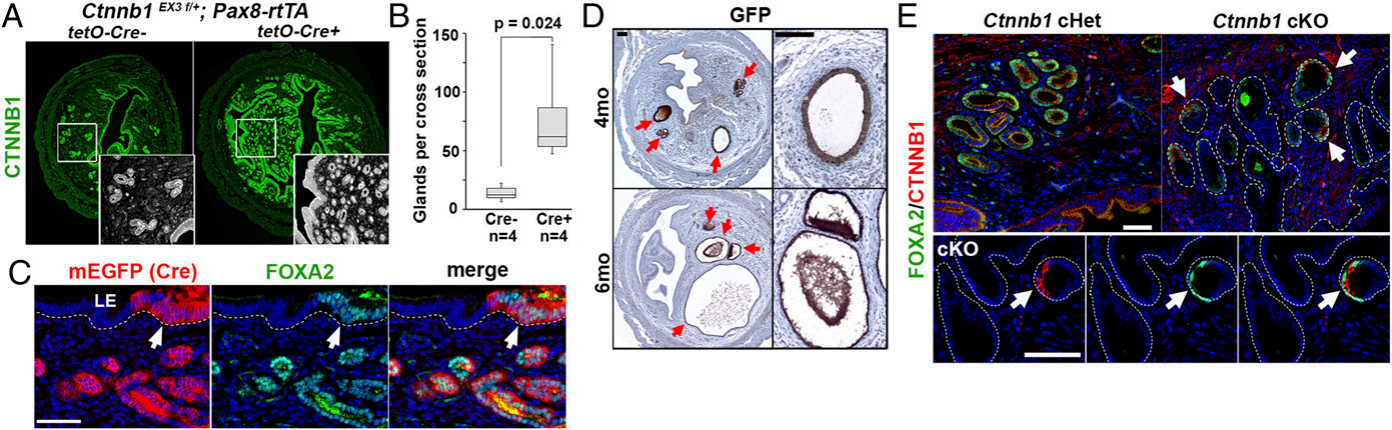
CTNNB1 exon 3 deletion promotes neoadenogenesis in the mature uterine epithelium. (*A*) The number of uterine glands per uterine cross-section was compared between CTNNB1 GOF mutant (Cre^+^) and control (Cre^−^) at PD60. (*B*) Boxplot presentation of uterine gland density in *Ctnnb1* exon 3 deletion mutant and control uteri. UtE-specific expression of mutant CTNNB1 in PD21 mice significantly increases the number of uterine glands at PD60 (*P* = 0.024). (*C*) Deletion of *Ctnnb1* exon 3 by Cre (detected by mEGFP reporter expression) induced FOXA2 expression (arrow) in the luminal epithelium (LE). Epithelial basement membrane is highlighted by a dotted line. (*D*) Sustained CTNNB1 activity in UtE resulted in nonproliferative cysts (red arrows). (*E*) FOXA2 and CTNNB1 expression in the luminal and glandular epithelium of *Ctnnb1* conditional heterozygous (cHet) and knockout (cKO) mice. In cKO mice, FOXA2 expression was detected only in glandular cells retaining CTNNB1 (arrows). (Scale bars: *C* and *D*, 50 μm; *E*, 100 μm.)

The restriction of mEGFP (Cre reporter)-positive cells within uterine glands (Fig. 1*A*) suggest that cells with CTNNB1 GOF could survive only in the uterine glands or could initiate neoadenogenesis, the formation of new glands. The critical importance of canonical WNT-CTNNB1 pathway in neoadenogenesis has been demonstrated in several different mutant mouse strains: The loss of *Wnt4* (24), *Wnt7a* (25–27), *Lef1* (28), or *Ctnnb1* (29) in uterine cells resulted in the absence of uterine glands. Furthermore, CTNNB1 stabilization in embryonic uterine epithelium or Müllerian duct mesenchyme increased the number of uterine glands per uterus (30, 31). In mice, neoadenogenesis is initiated around postnatal day (PD) 7, as epithelial cells positive for FOXA2, an essential transcription factor for uterine gland formation (32), grow into underlying stroma. Neoadenogenesis is completed by the second week of postnatal development (33). Adenogenesis in the mouse uterus continues thereafter with expansion of existing FOXA2-positive glandular epithelial cells (34–36). Thus, we tested if stabilization of CTNNB1 in fully differentiated UtE of prepubertal mice could initiate neoadenogenesis. When *Ctnnb1* exon 3 was deleted in the UtE of PD21 mice utilizing doxycycline-induced Cre transgenic lines (*Pax8-rtTA*; *tetO-Cr*e) (37), the number of uterine glands per section significantly increased (Fig. 3 *A* and *B*). In normal uteri, the expression of FOXA2 is restricted to the uterine glands. However, stabilization of CTNNB1 induced FOXA2 in the luminal epithelium (Fig. 3*C*), indicating that expression of mutant CTNNB1 in UtE is sufficient to activate the adenogenesis program in the uterus of PD21 mice. Hence, CTNNB1 mutant cells were found only in the uterine glands (Fig. 1*A*) because CTNNB1 mutation initiated adenogenesis. While CTNNB1 mutation alone did not induce histologically detectable abnormalities at 2 mo (Fig. 1*A*), continuous expression of mutant CTNNB1 resulted in nonproliferative cysts in 75% (3/4) and 100% (4/4) of mice at 4 and 6 mo, respectively (Fig. 3*D*).

As sustained CTNNB1 activity resulted in uterine gland dysfunction, we tested if CTNNB1 activity was required for the maintenance of uterine glands by ablating CTNNB1 with Lactoferrin-iCre (*Ltf*^*iCre*^), which is activated in the UtE after puberty (38). By 4 mo of age, FOXA2 expression gradually disappeared along with CTNNB1, as *Ltf*^*iCre*^ deleted *Ctnnb1*-floxed alleles (39). Although the structure of uterine glands remained, only a subset of glandular epithelial cells that retained CTNNB1 still expressed FOXA2 (Fig. 3*E*). Since FOXA2 is essential for the functions of uterine glands (40), cell-autonomous CTNNB1 activity is required for the maintenance of functional uterine glands.

### Synergistic Effects of CTNNB1 Mutation with PTEN and PI3K Mutations.

**Fig. 4.**
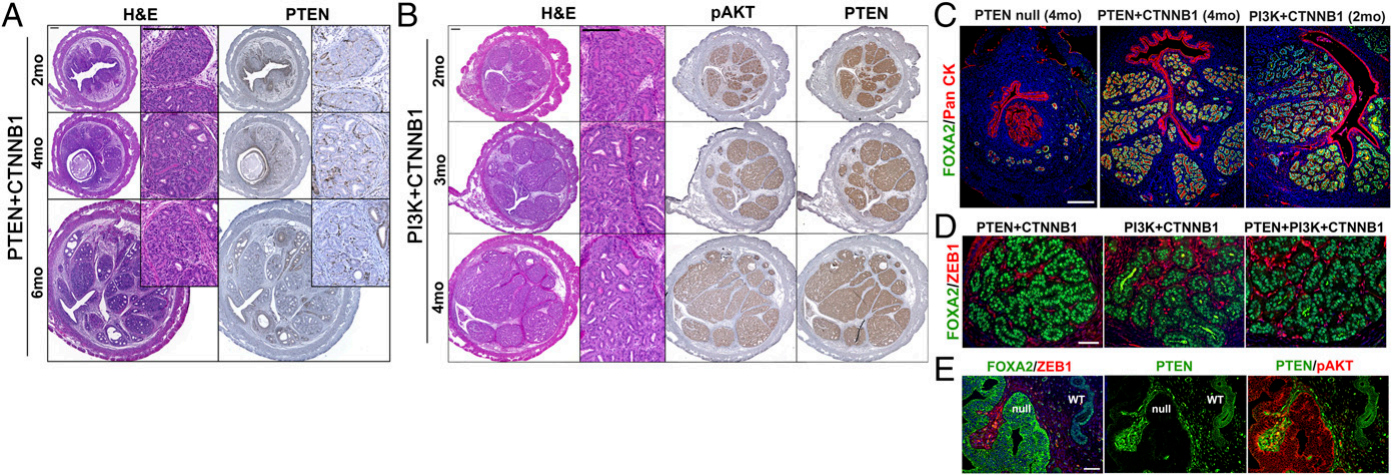
Development of glandular hyperplasia in the uteri from mice carrying the combination of CTNNB1 mutation with PTEN LOF or PI3K GOF mutation. Histopathological analyses of uteri carrying CTNNB1 GOF mutation with *Pten* biallelic loss (*A*) or PI3K GOF (*B*) mutation at indicated time points (months). The loss of PTEN (*A*) and the expression of pAKT (*B*) in epithelial lesions. (*C*) Glandular hyperplasia lesions were positive for uterine gland marker (FOXA2) and were organized in normal lobular structures. (*D*) Each gland in the hyperplastic lesions was separated by ZEB1-positive interstitial stromal cells. (*E*) FOXA2 was excluded from the nucleus of UtE with biallelic *Pten* loss. (Scale bars: *A*–*C*, 200 μm; *D* and *E*, 50 μm.)

Development of glandular hyperplasia associated with combinations of CTNNB1 mutation with PTEN and/or PI3K mutation (Fig. 1 *G*–*I*) is likely attributable to the neoadenogenesis activity of CTNNB1 mutation and the growth-promoting effects of PTEN and PI3K mutations. When the progression of PTEN+CTNNB1 mutant cells (Fig. 4*A*) and PI3K+CTNNB1 mutant cells (Fig. 4*B*) was followed for 6 and 4 mo, respectively, glandular hyperplasia increased by volume. Nevertheless, the phenotypes of epithelial lesions remained the same: The hyperplastic glands were always organized in the lobular structure of normal uterine glands (Fig. 4*C*), and each gland was surrounded by interstitial stromal cells (Fig. 4*D*).

Interestingly, PTEN-null (biallelic/single-gene) mutant cells never exhibited typical glandular morphology with nuclear FOXA2 expression in the analysis of over 80 lesions from 19 mice. Instead, cytoplasmic FOXA2 expression was detected in four PTEN-null lesions from four *Pten*^*f/f*^ mice (Fig. 4*E*). This observation suggests that PTEN-null mutation inhibits glandular differentiation by inducing phosphorylation and nuclear exclusion of FOXA2 by AKT (41). Nevertheless, PTEN-null cells could form glands when CTNNB1 mutation overrides the effect of PTEN LOF by overexpression of FOXA2 (30).

A single instance of well-differentiated EEC was seen in one of six PTEN+CTNNB1 mutant mice euthanized at 6 mo (*SI Appendix*, Fig. S3). There was only a single nodule in the mouse (*SI Appendix*, Fig. S3*A*). Given the low rate of neoplastic transformation, it seems probable that the acquisition of additional genetic and/or epigenetic changes to PTEN and CTNNB1 mutations is necessary for malignant degeneration.

### CTNNB1 Exon 3 Mutations Are Associated with Expression of Adenogenetic Genes in Human EECs.

In TCGA dataset (2), genes associated with canonical WNT pathways were enriched in CL-EECs positive for *CTNNB1* exon 3 mutations (*SI Appendix*, Fig. S4*A*). In addition, *FOXA2*, *LEF1*, *DLX5*, and *DLX6* (42), genes that are essential for uterine gland formation, were elevated with *CTNNB1* mutations (*SI Appendix*, Fig. S4 *B* and *C*). One of the most enriched genes in *CTNNB1* mutant EECs was runt-related transcription factor 2 (RUNX2). In mice, RUNX2 was enriched in subepithelial stroma and glandular epithelial cells during adenogenesis (*SI Appendix*, Fig. S5*A*). The expression pattern suggests that RUNX2 is involved in uterine adenogenesis as a downstream transcription factor of WNT-CTNNB1 pathways. Indeed, *Ctnnb1* exon 3 deletion induced RUNX2 in mouse UtE. As in human EECs, DLX5 was also enriched in CTNNB1 mutant hyperplastic lesions. Additionally, SOX17, a transcription factor essential for adenogenesis, was also enriched in the glandular hyperplasia (*SI Appendix*, Fig. S5*B*). These observations suggest that CTNNB1 exon 3 mutations activate neoadenogenesis in both human and mouse UtE.

### The Combination of PTEN, PI3K, and CTNNB1 Mutations Promotes Preneoplastic Transformation.

**Fig. 5.**
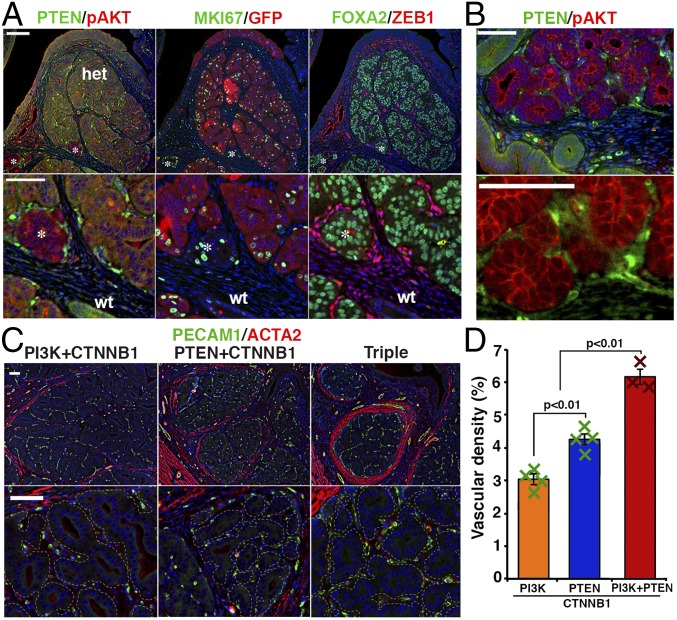
Gene-dose effect of PTEN, PI3K, and CTNNB1 mutations in uterine epithelial lesions. (*A*) In triple-gene mutant mice, dominant epithelial lesions expressed a low level of PTEN indicating monoallelic loss, EGFP (marker for PIK3CA* expression), and FOXA2 (markers for CTNNB1 mutation). Small clusters of PTEN-null, FOXA2-positive glands (PIK3CA* negative) were also detected (*). (*B*) Hyperplastic glands with PTEN mutation were closely associated with cells having intense PTEN immunostaining signals. Cellular morphology indicated they were endothelial cells. (*C*) Density and size of microvasculatures within hyperplastic lesions increased in triple-gene mutant mice compared with double-gene (PI3K+CTNNB1 and PTEN+CTNNB1) mutant mice. Microvasculatures are PECAM1-positive (green) and ACTA2-negative (red). Epithelial lesions were outlined with dotted lines. (Scale bars: 50 μm.) (*D*) Vascular density in the lobules of glandular hyperplasia in double-gene and tripe-gene mutant mice carrying the CTNNB1 mutant allele (2 mo). Result is presented by average ± SE.

All triple-gene mutant (PTEN+PI3K+CTNNB1) mice were euthanized by 75 d after Ad-Cre injection because of health issues due to blockage of the urinary tract by rapidly growing vulvar tumors. Within this experimental time frame, EEC never developed in these mice: The hyperplastic glands in triple-gene mutant mice were not neoplastic, as they were separated by stromal cells (Fig. 4*D*). The genotype of major epithelial lesions was monoallelic *Pten* loss + PI3K + CTNNB1 (Fig. 5*A*), and PTEN-null mutation and PIK3CA* expression were mutually exclusive. While PTEN+CTNNB1 double-gene mutant lesions were also detected in 100% of triple-gene mutant mice (Fig. 5*A*, *), they remained small, indicating a dose-effect of mutant genes, which explains the high rate of co-occurrence of *PTEN*, PI3K, and *CTNNB1* mutations in human EECs (*SI Appendix*, Fig. S4*D*) (2).

The hyperplastic glands of PTEN+CTNNB1 double-gene and PTEN+PI3K+CTNNB1 triple-gene mutant mice were always associated with stromal cells that were highly positive for PTEN (Fig. 5*B*). The morphology (Fig. 5*B*) and PECAM1 (Fig. 5*C*) expression indicated that these PTEN-positive cells were primarily endothelial cells. In addition, the lobules of glandular hyperplasia in double-gene and triple-gene mutant mice were surrounded by α smooth muscle actin (ACTA2)-positive cells (Fig. 5*C* and *SI Appendix*, Fig. S6). These observations suggest that the mutant epithelial cells within the glandular hyperplasia secrete factors that induce angiogenesis and myodifferentiation of fibroblasts (43, 44). Particularly, the density of microvasculatures associated with glandular hyperplasia increased as the number of mutant alleles increased (Fig. 5*D*), indicating the dosage-dependent effect of mutant alleles in the progression of endometrial epithelial lesions.

### Effect of Ovarian Insufficiency on EEC Progression.

**Fig. 6.**
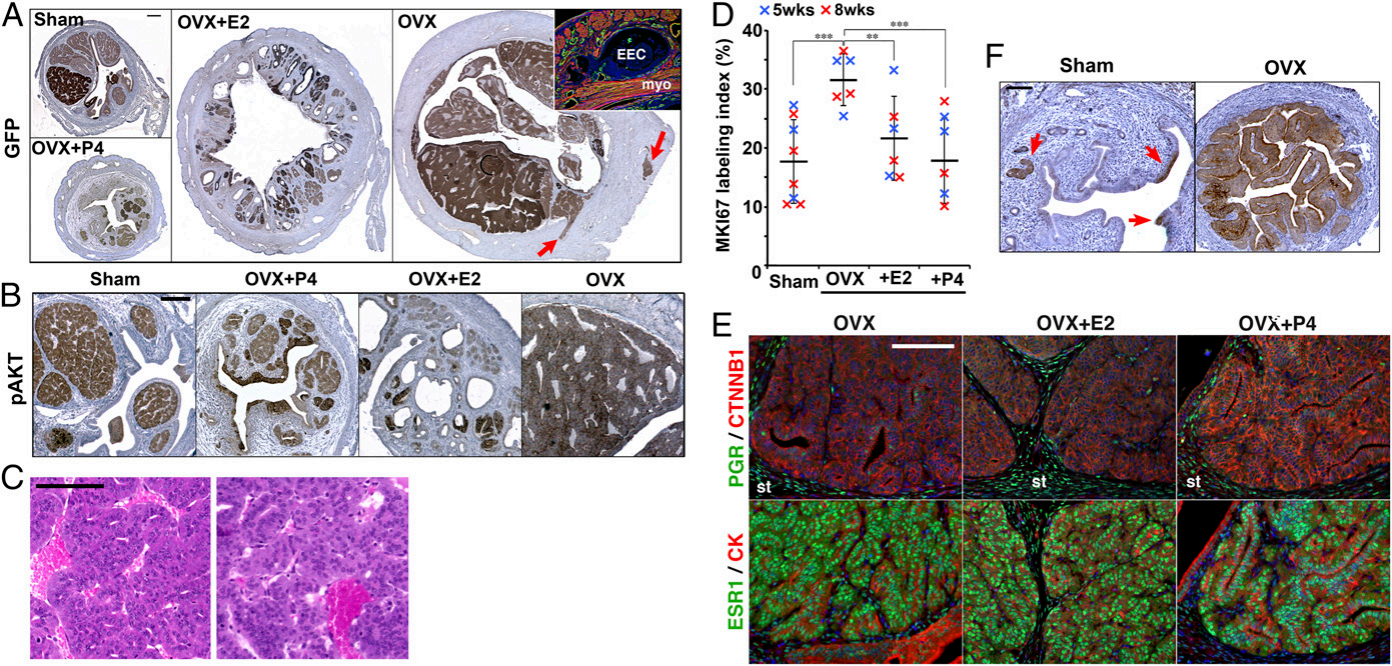
Steroid hormone deficiency promotes endometrial carcinogenesis. (*A* and *B*) The uterine pathology of triple-gene mutant mice was compared among sham-operated (Sham), ovariectomized (OVX), OVX+P4, and OVX+E2 groups at 8 wk after mutagenesis. (*A*) Uterine size and gross morphology of epithelial lesions (highlighted by GFP immunohistochemistry). Myoinvasive lesions are indicated by arrows. IF staining (red for ACTA2 and green for collagen IV) of a myoinvasive lesion in OVX group is shown in *Inset*. (*B*) Morphology of epithelial lesions (highlighted by pAKT immunohistochemistry). (*C*) Representative histopathology (H&E staining) of EECs in OVX group at 6 wk after ovariectomy. (*D*) Effect of E2 and P4 on epithelial MKI67 labeling index in glandular hyperplasia. Blue and red plots represent values at 5 and 8 wk after mutagenesis, respectively. The horizontal lines and vertical lines indicate average and SD, respectively. **P < 0.01, ***P < 0.001. (*E*) Effect of E2 and P4 on the expression of PGR and ESR1 in glandular hyperplasia of triple-gene mutant mice. St, stroma. (*F*) Effect of castration on PTEN+PI3K double-gene mutant lesions. pAKT immunohistochemistry at 6 wk after virus injection (4 wk after sham operation or ovariectomy) is shown. Red arrows in Sham group indicate pAKT-positive cells. (Scale bars: *A*, 200 μm; *B*–*D*. 100 μm.)

EEC usually presents in postmenopausal women. Although menopause is strongly associated with multiple factors that increase uterine cancer risk, including old age and loss of menstruation, the mechanisms through which menopause increases the risk for EECs remain unclear. In our mouse model, the effect of hormones on the progression of endometrial lesions can be tested independent of menstruation and aging effects. Thus, female triple-gene mutant mice were ovariectomized (OVX) or sham-operated (Sham) at 2 wk after Ad-Cre injection, and the OVX mice were supplemented with no hormone, 17β-estradiol (E2), or progesterone (P4) (45) (Table 2). The phenotypes of the Sham group were essentially identical to that of intact mice, demonstrating glandular hyperplasia by 2 mo (Fig. 6 *A* and *B*). Surprisingly, all OVX mice developed EEC within 2 mo after 10^9^ plaque-forming units (pfu)/mL Ad-Cre injection (Fig. 6 *A*–*C*). Remarkably, myometrial invasion was detected in 50% (2/4) at 5 wk and 83% (5/6) at 8 wk after Ad-Cre injection (Fig. 6*A*, OVX red arrows).

**Table 2.** Summary for hormonal effects on uterine pathology

Gene no.	Virus titer, pfu/mL	Treatment	Frequency of epithelial lesions, %							
			5–6 wk					8 wk		
			*n*	Lesions	Per mouse	Per section	*n*	Lesions	Per mouse	Per section
Triple	10^9^	Sham	7	Hyperplasia	100 (7/7)	88.8 ± 7.3	9	Hyperplasia	100 (9/9)	96.3 ± 2.5
		OVX	4	Hyperplasia	100 (4/4)	100.0 ± 0.0	6	Hyperplasia	100 (6/6)	100.0 ± 0.0
				EEC	100 (4/4)	97.0 ± 3.0		EEC	100 (6/6)	96.4 ± 3.6
				Myoinvasion	50 (2/4)	3.9 ± 2.6		Myoinvasion	83.3 (5/6)	14.4 ± 8.1
								Serosal metastasis	16.7 (1/6)	2.8 ± 2.8
		OVX+E2	3	Hyperplasia	100 (3/3)	91.7 ± 8.3	3	Hyperplasia	100 (3/3)	100.0 ± 0.0
				EEC	33.3 (1/3)	21.3 ± 21.3		EEC	66.7 (2/3)	29.5 ± 22.8
		OVX+P4	3	Hyperplasia	100 (3/3)	96.7 ± 3.3	3	Hyperplasia	100 (3/3)	95.2 ± 4.8
	10^8^	Sham	3	Hyperplasia	100 (3/3)	87.1 ± 3.3	4	Hyperplasia	100 (4/4)	87.5 ± 12.5
		OVX	4	Hyperplasia	100 (4/4)	100.0 ± 0.0	3	Hyperplasia	100 (3/3)	100.0 ± 0.0
				EEC	100 (4/4)	94.1 ± 6.0		EEC	100 (3/3)	100.0 ± 0.0
				Myoinvasion	50 (2/4)	8.6 ± 5.9		Myoinvasion	66.7 (2/3)	15.5 ± 7.8
								Serosal metastasis	33.3 (1/3)	2.4 ± 2.4
		OVX+E2	—	—	—	—	4	Hyperplasia	100 (4/4)	91.7 ± 8.3
								EEC	100 (4/4)	66.7 ± 21.2
		OVX+P4	3	Hyperplasia	100 (3/3)	84.9 ± 8.3	5	Hyperplasia	100 (5/5)	93.0 ± 7.0
Double	10^8^	Sham	4	Hyperplasia	0 (0/4)	0	4	Hyperplasia	0 (0/4)	0
		OVX	4	Hyperplasia	100 (4/4)	100.0 ± 0.0	4	Hyperplasia	100 (4/4)	100.0 ± 0.0
		OVX+E2	3	Hyperplasia	100 (3/3)	30.7 ± 21.8	2	Hyperplasia	50 (1/2)	42.9
		OVX+P4	3	Hyperplasia	0 (0/3)	0	3	Hyperplasia	33.3 (1/3)	9.38 ± 9.38

Five weeks for 10^9^ and 6 wk for 10^8^. Frequency of lesions per section is the percentage of uterine sections that contain the lesions in each mouse. It is expressed as mean ± SE among all mice in each group including mice that were negative for the lesion. In triple-gene mutant mice, per section frequencies of EEC, myoinvasion, and serosal metastatsis are significantly higher in OVX group than all other groups (*P* < 0.01).

Treatment with E2 or P4 that mimics serum hormone levels of cycling women (45) attenuated the progression of EEC in OVX mice (Fig. 6 *A* and *B* and Table 2), suggesting that the neoplastic transformation of glandular hyperplasia to EEC was due to deficiency of ovarian steroids. In the triple-gene mutant mice, OVX significantly increased the proliferation rate of epithelial cells within the glandular hyperplasia lesions, but E2 or P4 effectively attenuated the proliferation (Fig. 6*D* and *SI Appendix*, Fig. S7). Nevertheless, E2 and P4 did not completely block the growth of triple-gene mutant cells, and the size of hyperplastic lobules increased after ovariectomy even in the presence of E2 or P4 (*SI Appendix*, Fig. S8).

Progesterone receptor (PGR) was essentially absent in the nucleus of triple-gene mutant epithelial cells, whereas adjacent stromal cells expressed a high level of PGR (Fig. 6*E*). These observations suggest that P4 represses the progression of triple-gene mutant cells through stromal PGR. However, triple-gene mutant cells retained ESR1 (Fig. 6*E*). Hence, E2 may directly inhibit the proliferation of triple-gene mutant cells via epithelial ESR1. Alternatively, E2 may repress the growth of glandular hyperplasia through stromal ESR1, given E2 induced FOXO1 and pMAPK1/3 primarily in adjacent stromal cells (*SI Appendix*, Fig. S7).

The effect of hormone deficiency was tested in PTEN+PI3K double-gene mutant mice (Fig. 6*F* and Table 2). To detect the effect of castration and hormone treatment with higher sensitivity, we reduced the initial number of mutant cells by lowering the titer of Ad-Cre to 10^8^ pfu/mL (Fig. 6*F*). Using this titer, PTEN+PI3K double-gene mutant cells remained small patches within the normal epithelium for 2 mo (Fig. 6*F*). However, castration dramatically increased the size of epithelial lesions (Fig. 6*F* and Table 2), indicating that menopause can promote endometrial hyperplasia by allowing expansion of epithelial cells with no carcinogenic potential. However, the PTEN+PI3K double-gene mutant cells remained in the lumen of the uterus, underlining the significance of CTNNB1 mutations in the stromal invasion. In triple-gene mutant mice, two different titers of Ad-Cre (10^8^ and 10^9^ pfu/mL) made no significant difference in the uterine pathology (Table 2).

### Characteristics of Myometrial Invasion.

**Fig. 7.**
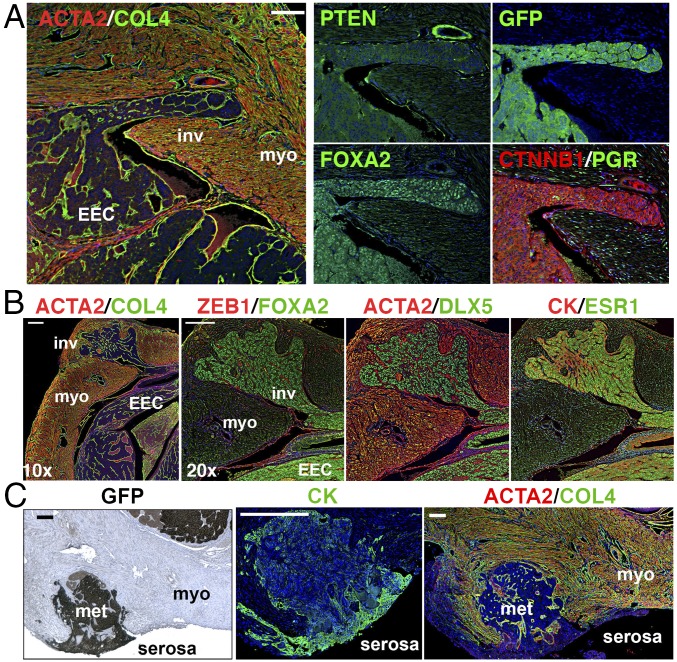
Characteristics of myoinvasive lesions and serosal metastasis. (*A*) Myometrial invasion of EECs at 8 wk after mutagenesis. The myoinvasion (inv) was continuous with EECs within endometrium, enclosed in a continuous basement membrane highlighted by collagen IV (COL4). IF for PTEN, EGFP, and FOXA2 indicated invasive lesions were triple-gene mutant. EECs were negative for PGR. (*B*) Expression profile of transcription factors (FOXA2, DLX5, and ESR1) was identical between invasive lesions and EECs within endometrium. (*C*) Serosal metastasis. Metastatic EEC (met) expressing GFP and CK penetrated the myometrium (myo, ACTA2 positive) and reached the uterine serosa. (Scale bars: *A* and *C*, 100 μm; *B*, 200 μm.)

In triple-gene mutant mice, myoinvasion was detected in more than one-half of the OVX group (11/17) within 2 mo (Table 2). Although the genotypes of uterine epithelial lesions in the triple-gene mutant mice were heterogeneous as assessed by immunofluorescence (IF) (Fig. 5*A* and *SI Appendix*, Fig. S9), myoinvasive lesions were always positive for FOXA2 and PIK3CA* with reduced PTEN expression (Fig. 7*A*), suggesting the combination of these three mutations is critical for the myometrial invasion of EECs. EECs in the endometrium and those invading myometrium were continuous with no apparent difference in morphology and marker expression (Fig. 7*B*), suggesting that myoinvasion is not driven by a small subpopulation of cells that acquired invasive phenotypes. Furthermore, myoinvasive lesions positive for GFP and cytokeratin (CK) penetrated through the myometrium, reaching the serosal surface of the uterus (serosal metastasis, Fig. 7*C* and Table 2). These observations suggest that the combination of three gene mutations (CTNNB1+PI3K+PTEN) and ovarian insufficiency are sufficient for the myometrial invasion and serosal metastasis of EECs.

## Discussion

In this study, we developed a mouse EEC model, in which multiple driver mutations are introduced into a small fraction of fully differentiated uterine epithelial cells. Utilizing this model, we demonstrated that the accumulation of multiple driver mutations is essential for endometrial epithelial cells to establish a preneoplastic lesion within the tumor-suppressing microenvironment of normal endometrium. Their collaborative functions in the development of preneoplastic lesions explain why *PTEN*, *PIK3CA*/*PIK3R1*, and *CTNNB1* mutations are detected in the majority of well-differentiated low-grade EECs, although these mutations are not associated with stage, grade, or clinical outcome of EECs (46).

In the endometrium of cycling women, PTEN-negative glands without pathogenic features are highly prevalent (47, 48), indicating the insufficiency of PTEN LOF in endometrial carcinogenesis. In our current study, simple hyperplasia was the most advanced epithelial lesion caused by *Pten*-null mutation, replicating the clinical observations in human patients. Previous studies also demonstrated that *Pten*-null mutation induced by direct injection of adenovirus Cre into the mouse uterus caused atypical hyperplasia but not gross uterine tumors (10, 49). In contrast, mouse model studies utilizing uterine tissue-specific Cre transgenes (e.g., *Pgr-Cre*, *Ksp-Cre*, *Sprr2f-Cre*) demonstrated that *Pten* mutation alone causes large uterine tumors (50–52). Our current study suggests that the overgrowth of *Pten* mutant cells is repressed by the surrounding normal cells and that the growth-repressing field effect of normal endometrium is lost when *Pten* is mutated in a large portion of uterine cells by Cre transgenes.

Joshi et al. (51) previously reported the haploinsufficiency of PTEN in the repression of PI3K-driven mouse uterine cancers: PIK3CA^E545K^ expression in the Müllerian duct epithelium caused pathological lesions in the adult uterus only when a copy of *Pten* was inactivated, and the biallelic loss of *Pten* in combination with PIK3CA^E545K^ caused ECs. In our current study, PIK3CA* expression induced endometrial hyperplasia only when one *Pten* allele was inactivated, confirming the study by Joshi et al. However, in our mouse model mutant cells that harbored biallelic loss of *Pten* in combination with PI3K activation failed to persist in the adult uterus. The discrepancy between this and the previous studies is not surprising given the fundamental differences between the physiologies of uterus and Müllerian duct. Because of the highly proliferative and undifferentiated nature of the Müllerian duct, the tissue environment is expected to be permissive for the growth of cells containing a high level of PIP3. The absence of triallelic (PTEN-null+PI3K) mutant lesions may indicate a limitation of our mouse model, in which multiple mutant alleles are concurrently induced. In human endometrial carcinogenesis, cancer cells evolve through multiple cell divisions by stepwise accumulation of genetic and epigenetic alterations. Preneoplastic cells may tolerate the combination of PTEN-null and PI3K-activating mutations when these mutant alleles are acquired sequentially. In addition, the gradual progression of preneoplastic lesions would be accompanied with changes in the microenvironment, which should be permissive for the survival and growth of cells harboring PTEN-null + PI3K-activating mutations, as they are surrounded by mutant epithelial cells.

Our current study demonstrated that the *CTNNB*1 mutation in exon 3 is sufficient to drive neoadenogenesis in mature endometrium, and that the continuous presence of CTNNB1 activity is essential for the maintenance of functional uterine glands. Uterine neoadenogenesis involves reciprocal interactions between epithelial and stromal cells (53). Thus, *CTNNB1* exon 3 mutations should confer endometrial epithelial cells an ability to grow into the stroma through paracrine communications with stromal cells. In addition, the *CTNNB1* exon 3 mutation promotes the myoinvasion of EECs in collaboration with ovarian insufficiency. In human EECs, the depth of myometrial invasion is positively correlated with the risk of recurrence (54), and the risk of recurrence of early stage EECs increases sixfold with *CTNNB1* mutations (8). The results of our mouse study connect these two observations by establishing the role of *CTNNB1* mutation in the myoinvasion of EECs. Accordingly, *CTNNB1* exon 3 mutations play a central role in the recurrence of stage I EECs. Liu et al. (55) proposed clustering of 271 EC cases from TCGA dataset into four subgroups by the transcriptome profile, and *CTNNB1* mutant cancers were enriched in one of four subgroups. Interestingly, the average age of first diagnosis in the subgroup with a high *CTNNB1* mutation rate was significantly younger than the other three groups. We speculate that the early diagnosis of *CTNNB1* mutant EECs is due to the activation of the adenogenesis program, allowing mutant cells to propagate into the stratum basalis, where they are protected from removal by menstruation. In the light of the adenogenesis theory, the recurrent mutations of *SOX17* (56, 57) and *FOXA2* (58) in EECs (*SI Appendix*, Fig. S4*D*) are probably not a coincidence.

This study experimentally demonstrated the necessity of multiple genetic alterations for the development of preneoplastic epithelial lesions in the uterus. Loss of PTEN protein is the one of most common initial events in the pathogenesis of EECs (48, 59, 60). However, growth potential of *PTEN* mutant cells in normal endometrium of cycling women is limited, and acquisition of additional driver mutations, such as *PIK3CA*/*PIK3R1* or *CTNNB1*, is essential for development of preneoplastic lesions. Particularly, acquisition of *CTNNB1* exon 3 mutations would have significant impact in the progression of preneoplastic cells, as it promotes the spread of mutant cells into the stroma.

As confirmed by this study, endometrial carcinogenesis is a multistep process driven by compounding genetic events. Since the growth of normal endometrial epithelial cells depends on estrogens, estrogen exposure should elevate EC risk by increasing the chance for the acquisition of EC-initiating mutations. However, there is no evidence that estrogen promotes the subsequent phases/steps of endometrial carcinogenesis. Our current mouse model study concludes that E2 represses the progression of initiated (mutant) epithelial cells. In this mouse model, E2 effects on EC progression can be evaluated independent of E2 effects on the initiation, as EC-initiating mutations are induced by Ad-Cre. Therefore, in epidemiological studies, the EC-initiating effects of estrogen may have masked the EC-repressing effects. Additionally, the hormone pellets used in this study sustain systemic E2 and P4 at the peak levels of cycling women, which are severalfold higher than peak levels of female mice. Thus, it is possible that estrogen at a dose higher than physiological levels is required to inhibit the progression of preneoplastic cells. The levels of estrogens in women with hormone-replacement therapies may also not be sufficient to repress the progression of EECs. It remains unanswered if a physiological level of E2 or P4 alone can repress the progression of EECs. Nonetheless, it is clear that ovarian activities repress the progression of preneoplastic mutant cells into EECs. Thus, we propose that homeostasis of the endometrium is gradually lost in middle-aged women as ovarian activities wane, allowing the overgrowth of mutant epithelial cells, which increases the chance to obtain additional alterations leading to malignant transformation. Most importantly, ovarian insufficiency allows myometrial invasion of mutant epithelial cells that harbor a *CTNNB1* exon 3 mutation.

Our mouse model replicated the clinical potential of P4 in repressing the growth of hyperplastic lesions (61–63). Since triple-gene mutant cells were mostly negative for PGR, P4 likely represses endometrial carcinogenesis by acting through stromal cells as found in the normal uterus (64–69). Additionally, normal epithelial cells adjacent to mutant epithelial cells appear to play a critical role in repressing the progression of EECs because aggressive epithelial lesions developed when *Pten* was mutated in the entire uterine epithelium by *Ltf*^*iCr*e^ (70). To develop preventive and therapeutic treatments for EECs, it is crucial to elucidate the molecular mechanisms underlying the tumor-repressing effects of the normal endometrial environment and steroid hormones.

## Methods

### Animals.

All experiments were performed under the Ohio State University Institutional Animal Care and Use Committee, approved protocols 2014A00000064. Mice were housed and bred in a barrier facility and were provided with food and water ad libitum. The mouse strains carrying alleles were used as follows: *ROSA*^*TE*^ (Gt*(ROSA) 26Sor*^*tm4(ACTB-tdTomato,-EGFP)Luo*^*/J*) (17), *Pten*^*f*^ (B6.129S4-*Pten*^*tm1Hwu*^*/J*) (14), *ROSA*^Pik3ca*^ (Gt*(ROSA)26Sor*^*tm7(Pik3ca,EGFP)Rsky*^*/J*) (15), *Ctnnb1*^*f(ex3)*^ (*Ctnnb1*^*tm1Mmt*^) (16), *Ctnnb1*^*tm1Knw*^*/J* (39), and *Ltf*^*iCre*^ (*Ltf*^*tm1(icre)Tdku*^) (38). All original strains were backcrossed to C57BL/6J mice (Jackson Laboratory) at least five times before generation of compound mutant strains. PTEN LOF allele was generated by deletion of exon 5 in the *Pten*^*f*^ allele. GOF mutations of PI3K were induced by expression of PIK3CA*, a constitutively active form of PI3K that consists of PIK3CA (p110α) and the iSH2 domain of PIK3R1 (p85α) connected by a flexible hinge region of a glycine linker. When Cre removes the lox-stop-lox cassette, the *ROSA* endogenous promoter drives the expression of PIK3CA* and EGFP (15). The effect of missense mutations in exon 3 (*SI Appendix*, Fig. S4*A*) was mimicked by the deletion of *Ctnnb1* exon 3 in *Ctnnb1*^*f(ex3)*^ allele (16). In some experiments, Cre-mediated gene recombination was monitored by the expression of mEGFP from *ROSA*^*TE*^ allele (17, 71), as specified.

### Adenoviral iCre-Mediated Mutagenesis.

Adenovirus vector for iCre recombinase (Ad-Cre) was purchased from Vector Biolabs. Original virus stock solution was diluted with 12 mM ethylene glycol tetraacetic acid (EGTA) in hypotonic buffer [10 mM 4-(2-hydroxyethyl)-1-piperazineethanesulfonic acid (Hepes, pH 7.9), 1.5 mM magnesium chloride, 10 mM potassium chloride, 0.5 mM DTT and 0.2 mM phenylmethylsulfonyl fluoride] to 1× 10^7^, 10^8^, and 10^9^ pfu/mL (72). The day of birth was counted as PD1, and Ad-Cre was inoculated on the day of weaning (∼PD21). To avoid stromal infection through a disrupted epithelial layer by direct uterine injection, viral suspension (30 μL per mouse) was infused into uterine lumen through vaginal cavity by injecting at the future vaginal orifice with 31G insulin syringes (Becton, Dickinson and Company) (*SI Appendix*, Fig. S1*A*). Based on the titration experiments (*SI Appendix*, Fig. S1 *B*–*D*), 1 × 10^9^ pfu/mL was used for most experiments, but 1 × 10^8^ pfu/mL Ad-Cre was also used in some experiments as specified in the text. Because Ad-Cre injection through the future vaginal orifice (71) induced vulvar tumors in mice carrying the *ROSA*^Pik3ca*^ allele (*SI Appendix*, Fig. S1 *E* and *F*), the progression of endometrial lesions was followed up to 4 mo by when the vaginal cavity and urinary tract were obstructed by vulvar tumors.

### Doxycyclin-Induced *Ctnnb1* Exon 3 Deletion.

To develop doxycycline (DOX)-induced *Ctnnb1* exon 3 deletion in the UtE, mice carrying *Pax8-rtTA* (73); *tetO-Cre* (74) were crossed with *Ctnnb1*^*f(ex3)/f(ex3)*^;*ROSA*^*TE/TE*^ mice. Littermates were fed with water containing 500 μg/mL DOX hydrochloride hemiethanolate hemihydrate (Sigma-Aldrich) ad libitum for 2 wk after weaning (37). Triple heterozygous mice (*Ctnnb1*^*f(ex3)/w*^*; Pax8-rtTA; tetO-Cre*) were used as *Ctnnb1* exon 3-deleted mutants (Cre^+^), and tetO-cre–negative mice (*Ctnnb1*^*f(ex3)/w*^*; Pax8-rtTA*) were used as the controls (Cre^−^). Tissues were harvested at PD60. Without DOX treatment, the uterine histology of Cre^+^ and Cre^−^ mice were indistinguishable from that of wild-type mice.

### Hormonal Manipulation of EEC Model Mice.

In the hormone manipulation study, mice were subjected to ovariectomy or sham surgery at 2 wk after viral injection (PD35–38). The OVX mice were divided into three groups and supplemented with a slow releasing pellet of E2 or P4, or no pellet. s.c. implantation of E2 and P4 pellets sustains serum E2 and P4 levels of 374.5 pg/mL (*n* = 20, 95% CI 291.5–457.5) and 50 ng/mL (*n* = 20, 95% CI 37–63), respectively, for 2 mo (45, 75), which are equivalent to the peak E2 and P4 serum levels of cycling women. Uterine pathology was analyzed at 5–8 wk after viral injection.

### Histopathological Analysis.

Due to the heterogeneity at the cellular level, genotypes and phenotypes of epithelial lesions were assessed by immunofluorescence and immunohistochemistry, as described (76, 77). The concentration and the manufacturers of primary antibodies used for immunostaining are listed in *SI Appendix*, Table S1. Uterine horns were cut into three to five pieces, fixed with Modified Davidson’s fixative solution (Electron Microscopy Sciences), and processed into paraffin blocks. Pathological analysis was blindly performed with hematoxylin and eosin (H&E)-stained sections, adapting routine diagnostic criteria (78). Proliferative irregularities, such as crowding, budding, and cell stratification of glands, are classified as hyperplasia. ECs were identified as having back-to-back glands without intervening stroma. Severe nuclear atypia was absent in all samples analyzed. The presence of epithelial lesions was first assessed in ∼15 H&E-stained sections per uterine horn. Sections were ≥40 µm apart from each other. The adjacent slides of H&E-stained slides were then analyzed by immunostaining for EGFP, PTEN, pAKT, and CTNNB1 to detect the loss and gain of protein expression. Since the nuclear accumulation of mutant CTNNB1 was not distinctive, the expression of FOXA2 was also used to detect CTNNB1 exon 3 deletion. When no mutant (EGFP-positive and/or PTEN-null) cells were detected in the initial screening, additional sections that were at least 50 sections (>250 µm) apart from the section used for the initial screening were examined. When no EGFP-positive and/or PTEN-negative cells were found in >20 sections from the same uterine horn, it was considered as unsuccessful viral inoculation, and the animal was excluded from the analysis. The incidence of lesions per section was calculated as the percentage of sections that contain lesions based on the analysis of 8–42 sections per mouse. The effect of hormone on cell proliferation was determined by counting total and MKI67-labeled epithelial cells in the glandular hyperplasia (75). For the MKI67-labeling index, 523–2,433 (average 1,319) cells per sample and 6,982–10,349 (average 8,572) cells per group were counted in six to eight mice per group. Vascular density was measured in four PECAM1 stained uterine sections from three to four mice per genotype. Lobules of glandular hyperplasia lesions (4–12 lobules per section) were manually selected, and the numbers of total and PECAM1-positive pixels (average 6.5 × 10^6^ pixel per mouse, excluding luminal space of glands) were counted utilizing ImageJ (NIH). Vascular density was calculated as the percentage of PECAM1-positive pixels within hyperplastic lobules in each mouse.

### Statistical Analysis.

Statistical significance was analyzed by one-way ANOVA with post hoc Tukey’s honestly significant difference (HSD) test for multiple comparison or Student’s *t* test for paired comparison. For multiple comparisons of *n* ≥ 10, statistical significance was further confirmed by the Bonferroni–Holm multiple comparison methods.

## Supplementary Material

Supplementary File
