# Supplementary material for: Ovarian insufficiency and CTNNB1 mutations drive malignant transformation of endometrial hyperplasia with altered PTEN/PI3K activities
Source: Proc Natl Acad Sci U S A. 2019 Feb 19;116(10):4528–37. doi: 10.1073/pnas.1814506116 (PMC6410785; doi:10.1073/pnas.1814506116)
Supplement: Supplementary File [file pnas.1814506116.sapp.pdf]

Table S1. Antibodies and applications

Antibody	Source	Catalog number	Use	Dilution
ACTA2	Abcam	ab7817	IF	1:500
COL4A	Abcam	ab19808	IF	1:200
FOXA2	Abcam	ab40874	IF	1:2,000
FOXO1	Abcam	ab179450	IF	1:100
GFP	Abcam	ab6673	IHC/IF	1:100
MKI67	Abcam	ab16667	IF	1:100
PECAM1	Abcam	ab28364	IF	1:200
RUNX2	Abcam	ab192256	IF	1:200
ZEB1	Abcam	ab180905	IF	1:1000
PAX2	Biolegend	PRB-276	IF	1:100
Phospho-AKT (Ser473)	Cell Signaling Technology	#4060	IHC/IF	1:100
Phospho-p42/44 MAPK (MAPK1/3) (Thr202/Tyr204)	Cell Signaling Technology	#4370	IF	1:30
PTEN	Cell Signaling Technology	#9188	IHC/IF	1:100
PGR	Dako	A0098	IF	1:200
DLX5	Proteintech	10592-1-AP	IF	1:100
SOX17	R&D Systems	AF1924	IF	1:500
pan-Cytokeratin (Pan CK)	Santa Cruz Biotechnology	sc-81714	IF	1:100
ESR1	Thermo Fisher Scientific	RM9101	IF	1:100
CTNNB1	Thermo Fisher Scientific	13-8400	IF	1:100

IHC: immunohistochemistry, IF: immunofluorescence

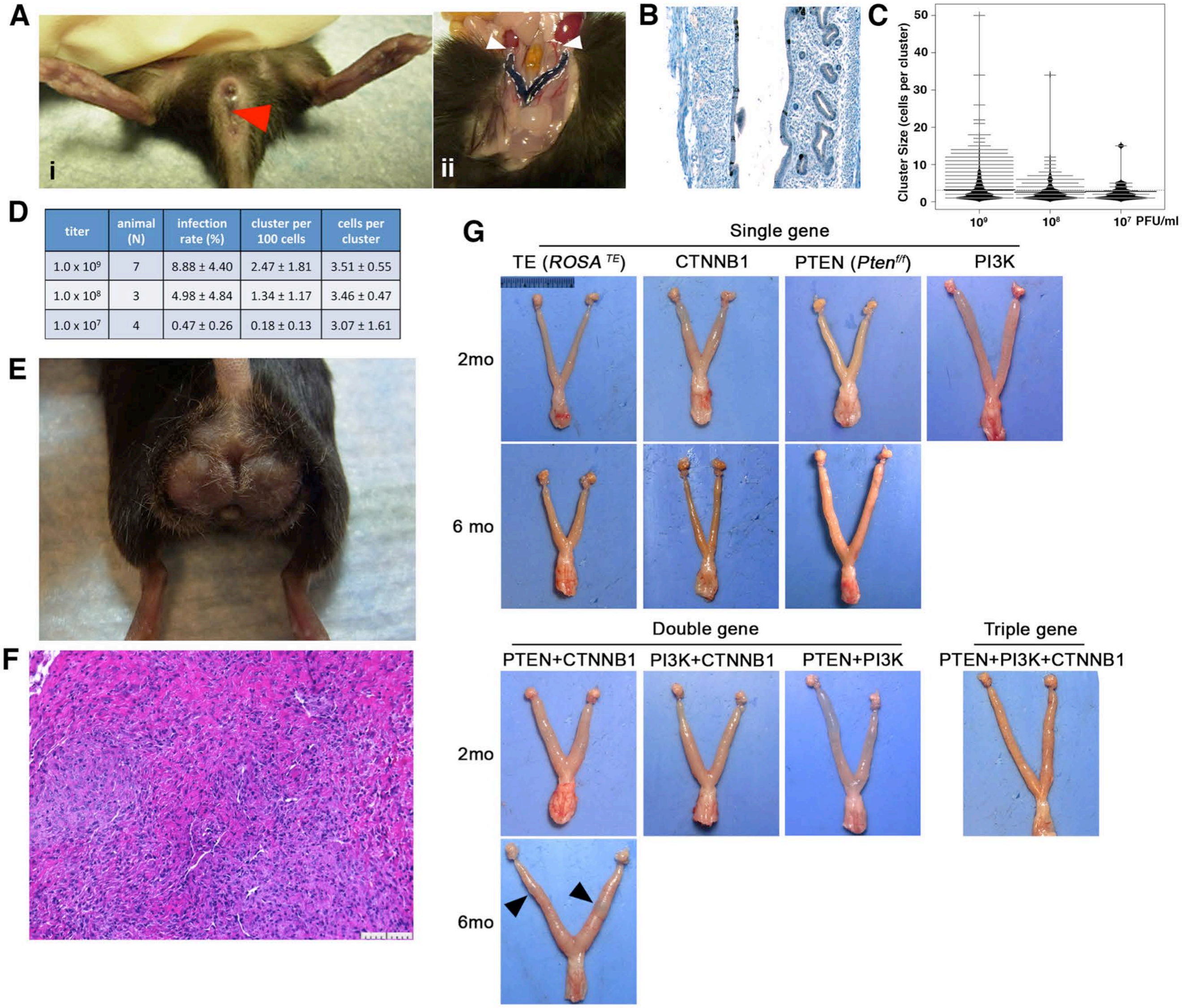

Fig. S1. Adenovirus-driven *in vivo* mutagenesis.

(A) Ad-Cre suspension was injected into the uterine lumen through the vaginal cavity. The red arrowhead (Ai) indicates the injection point, the unopened future vaginal-orifice. Trypan blue-PBS-filled uterine horns are outlined by dotted lines. The ovaries are indicated by white arrowheads (Aii). Trypan blue-PBS was used to visualize uterine horns for presentation purposes only. (B) Ad-Cre activity was visualized by immunohistochemistry for mEGFP reporter at 7 days after mutagenesis (representative image of 10^9 PFU/ml injected uteri). (C and D) Titration of Ad-Cre by recombination efficacy. The efficiency of 3 titers was compared by counting mEGFP positive cells in PTEN, PI3K and CTNNB1 triple-gene mutant (*Pten*$^{f/f}$; *Ctnnb1*$^{f(ex3)}$; *ROSA*$^{Pik3ca*/TE}$) mice a week after Ad-Cre injection. (C) Beanplot for the Ad-Cre titer and cluster size (cells per cluster) (D) Table for the titration test. Although the titer was 10 and 100-fold different, the distribution of mutant cell cluster size was similar among 3 titers. To achieve a high recombination efficiency/multiplicity of infection, 1x 10^9 PFU/ml was used for most experiments. (E and F) Vulva tumors developed in mice carrying *ROSA*^{Pik3ca*} at 4 months after *in vivo* mutagenesis. (E) Gross appearance and (F) H&E stained histology. (G) Macroscopic observation of the uteri from mice with indicated alleles at 2 and 6 months after *in vivo* mutagenesis. PTEN indicates *Pten*$^{f/f}$ genotype. There was no gross tumor detected within 2 months, however at 6 months epithelial lesions were occasionally detected by gross examination as a dilated segment due to blockage of the uterine canal by hyperplasia (black arrowheads).

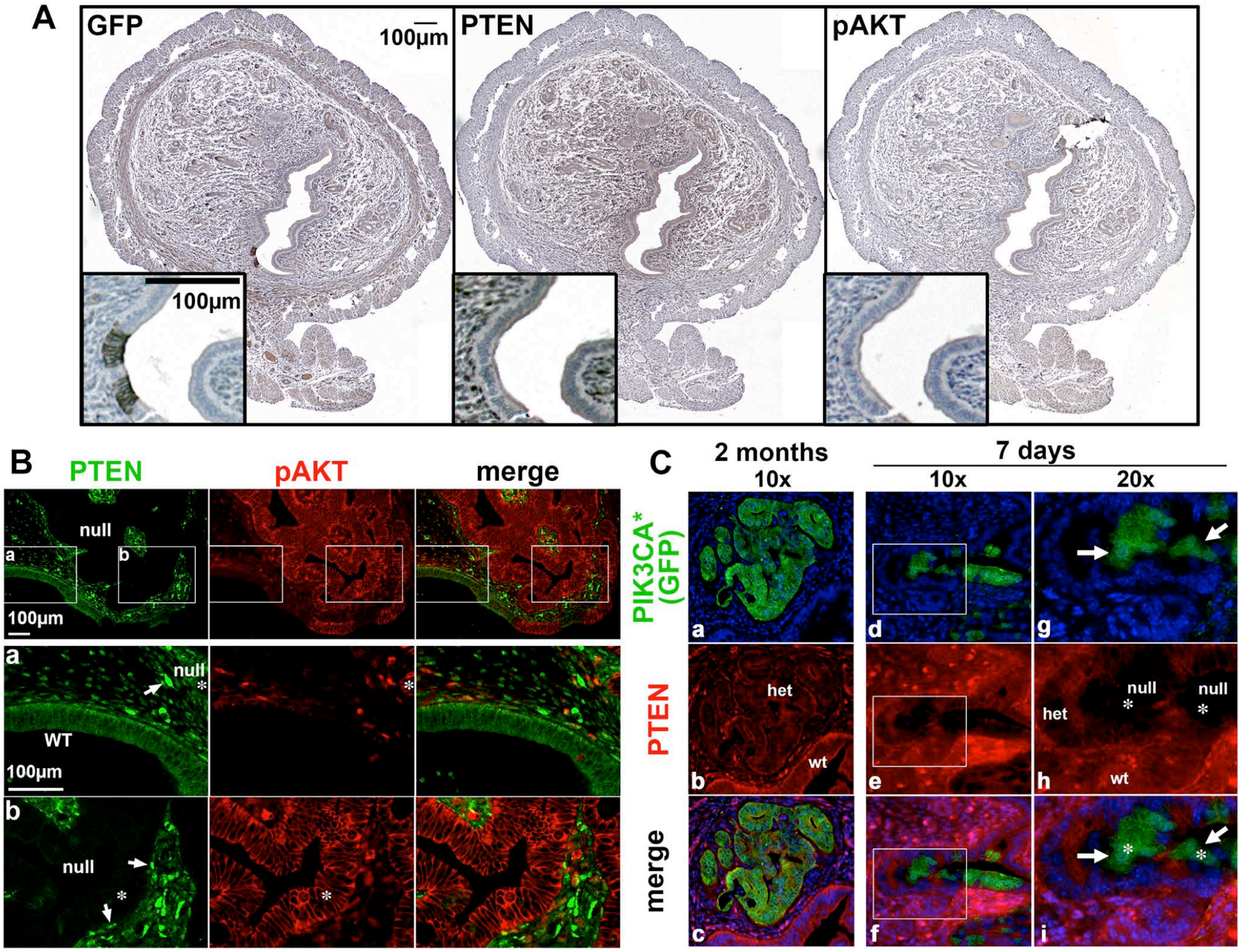

Fig. S2. Expression of PTEN, pAKT and PIK3CA* in mutant uterine epithelial cells.

(A) Monoallelic loss of *Pten* (*Pten*$^{f/w}$) does not alter epithelial phenotype in the mouse uterus. Cre-positive (mEGFP-positive) cells were indistinguishable from normal UtE. (B) Loss of PTEN in the uterine epithelium coincided with elevated pAKT in the epithelium and up-regulation of PTEN in the sub-epithelial stroma. Arrows indicate stromal cells with elevated PTEN expression. Asterisks indicate PTEN null cells. (C) Co-occurrence of biallelic *Pten* loss and PI3K GOF mutations was detected in the UtE at 7 days but not at 2 months after *in vivo* mutagenesis in the uteri of *Pten*$^{f/f}$; *ROSA*^{Pik3ca*} mice. At 2 months (a-c), double-gene/tri-allelic mutation (PTEN null + PIK3CA*-positive) was never detected. Most epithelial lesions were GFP-positive and weakly stained for PTEN, indicating expression of PIK3CA* and monoallelic loss of *Pten* (het). In contrast, there were PTEN-null cells (*) with GFP expression (arrows) at day 7 (d-i).

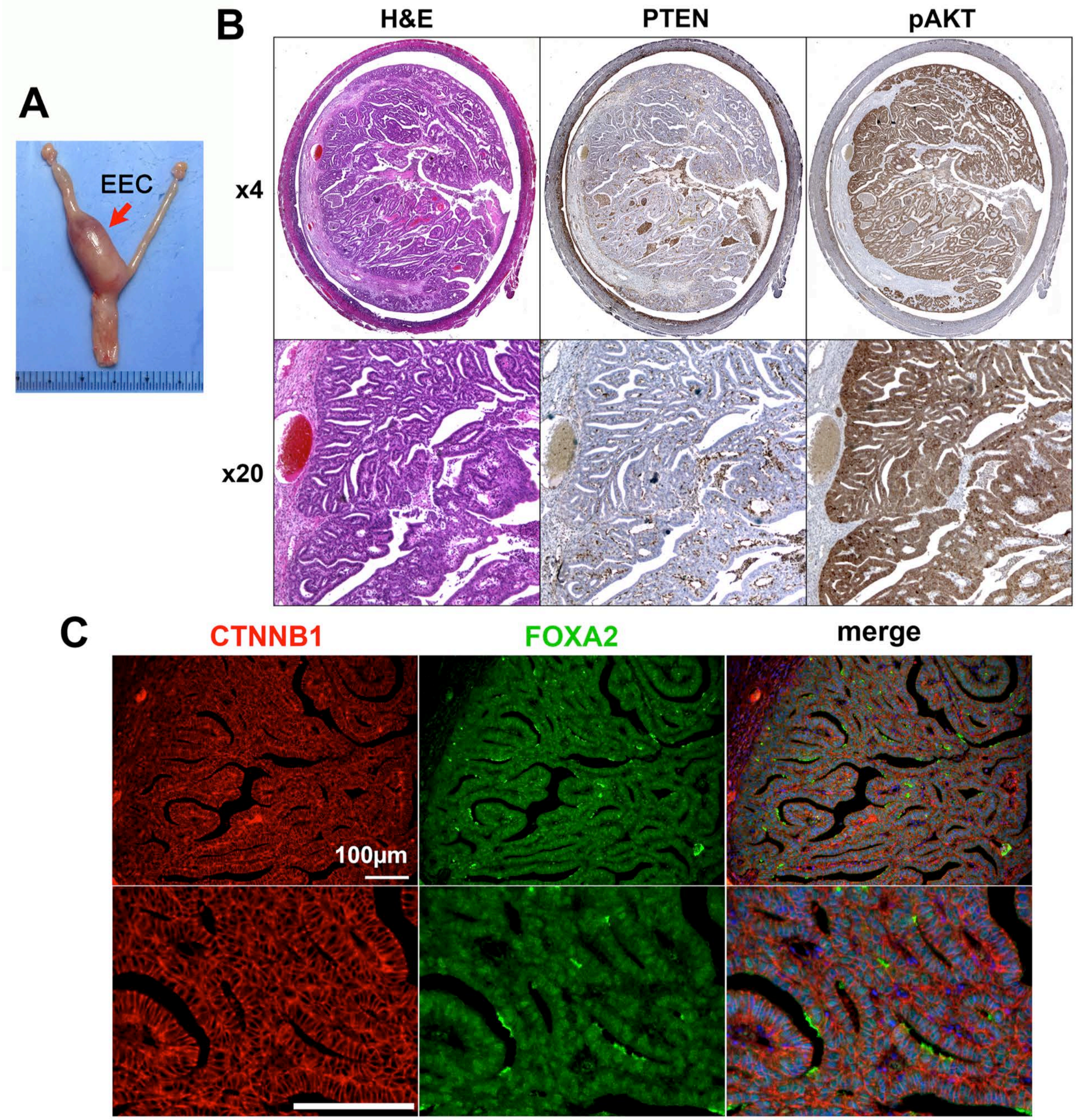

Fig. S3. Well-differentiated EEC observed in PTEN-null+CTNNB1 GOF mutant mice at 6 months after *in vivo* mutagenesis.

(A) Macroscopic image of the uteri. Tumor is indicated by an arrow. Thin uterine horns suggest potential hormonal deficiency of the mice. (B) Histopathology of EEC at a low magnification revealed no myometrial invasion. The EEC was negative for PTEN with elevated pAKT. (C) Immunofluorescence for CTNNB1 and FOXA2. Expression of FOXA2 throughout the EEC indicates the presence of CTNNB1 exon 3 mutation.

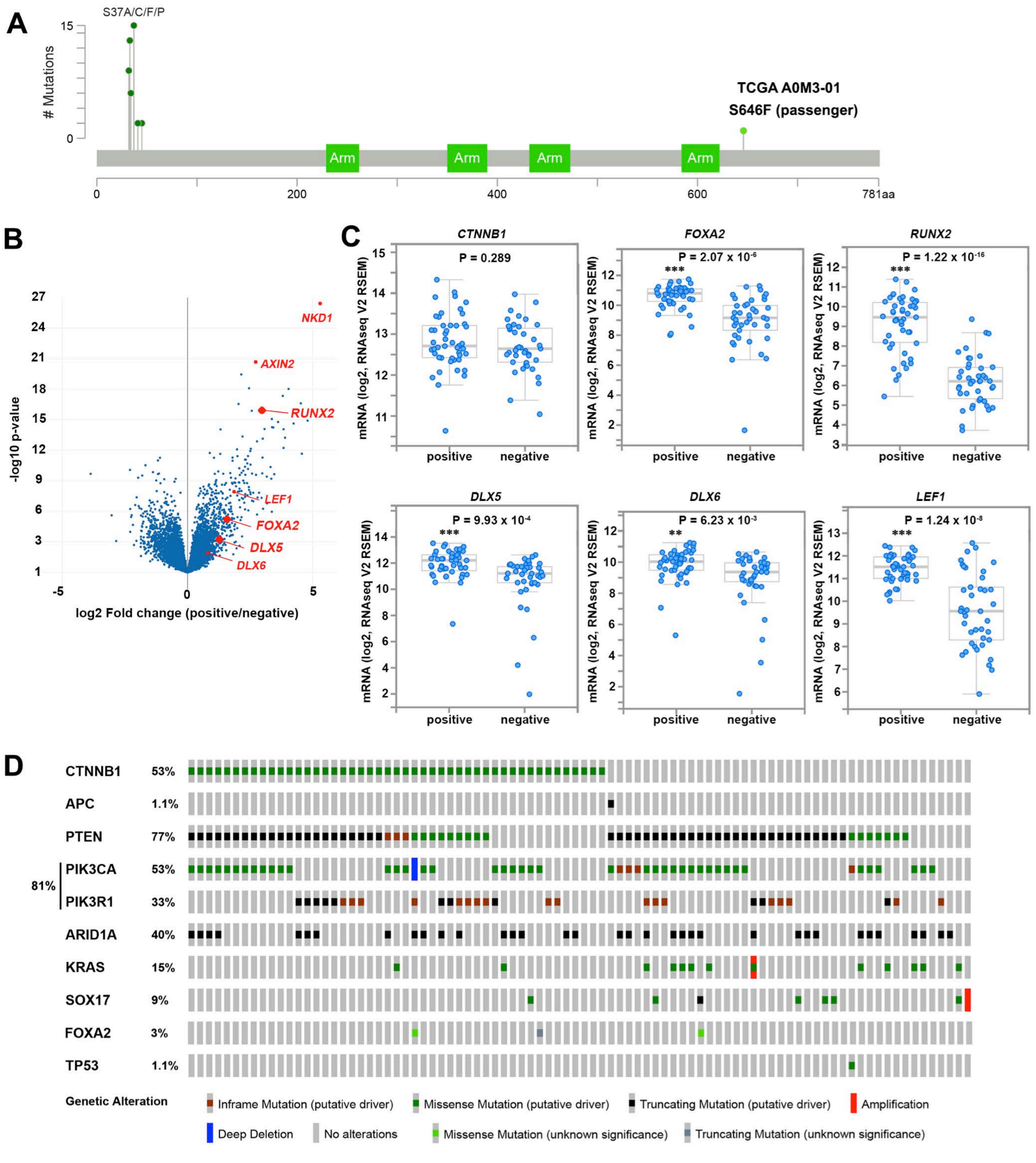

Fig. S4. Analysis of 88 CL-EECs in TCGA data set.

(A) The lollipop diagram of *CTNNB1* mutations in 88 CL-EEC cases. One case carried S646F, which is a putative passenger, and also harbored a S37F missense mutation. (B and C) Enrichment analysis of RNA-seq data by *CTNNB1* exon 3 mutation status (47 positive and 41 negative cases). (B) Genes associated with canonical WNT pathways and/or uterine gland formation are marked in the volcano plot. (C) Expression levels of genes transcripts were compared between *CTNNB1* exon 3 mutation positive and negative cases. (D) Oncoplot display of 88 CL-EEC cases. *TP53* mutation, which is common in serous-like ECs, was detected only in 1 case of CL-EECs. Mutations of unknown significance are shown only for *FOXA2*, as the functions of *FOXA2* mutations are unknown.

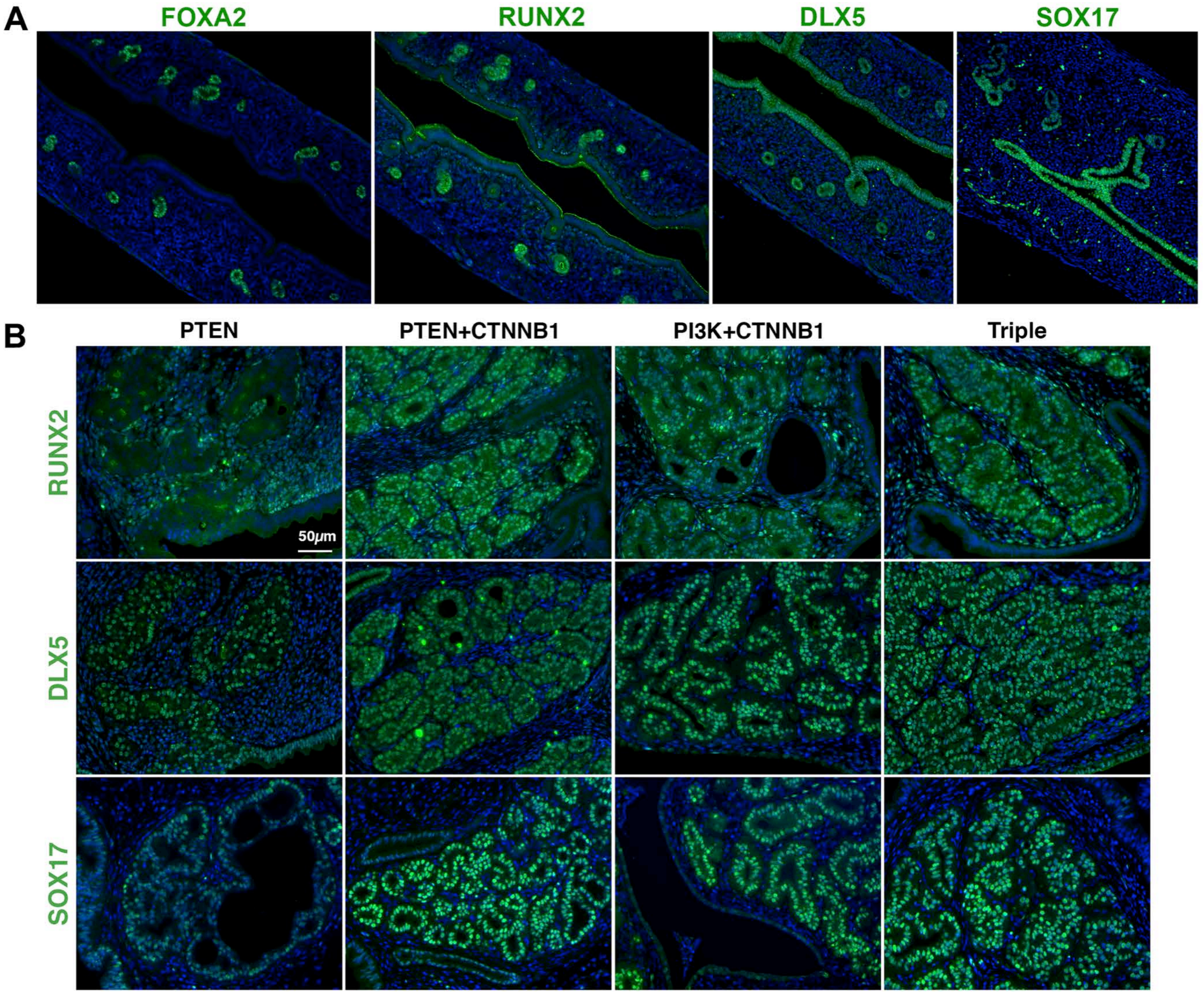

Fig. S5. Immunofluorescence assay for transcription factors essential for uterine adenogenesis.

(A) Expression of FOXA2, RUNX2, DLX5 and SOX17 in PD14 uteri. FOXA2 and RUNX2 are enriched in the glandular epithelium. DLX5 and SOX17 are uniformly expressed throughout the epithelium. (B) Expression patterns of RUNX2, DLX5 and SOX17 in hyperplastic lesions with different genotypes. RUNX2 is highly elevated in hyperplastic glands with CTNNB1 exon 3 mutation. DLX5 and SOX17 are also moderately enriched in glandular hyperplasia, but their expression was also detected in normal luminal epithelial cells.

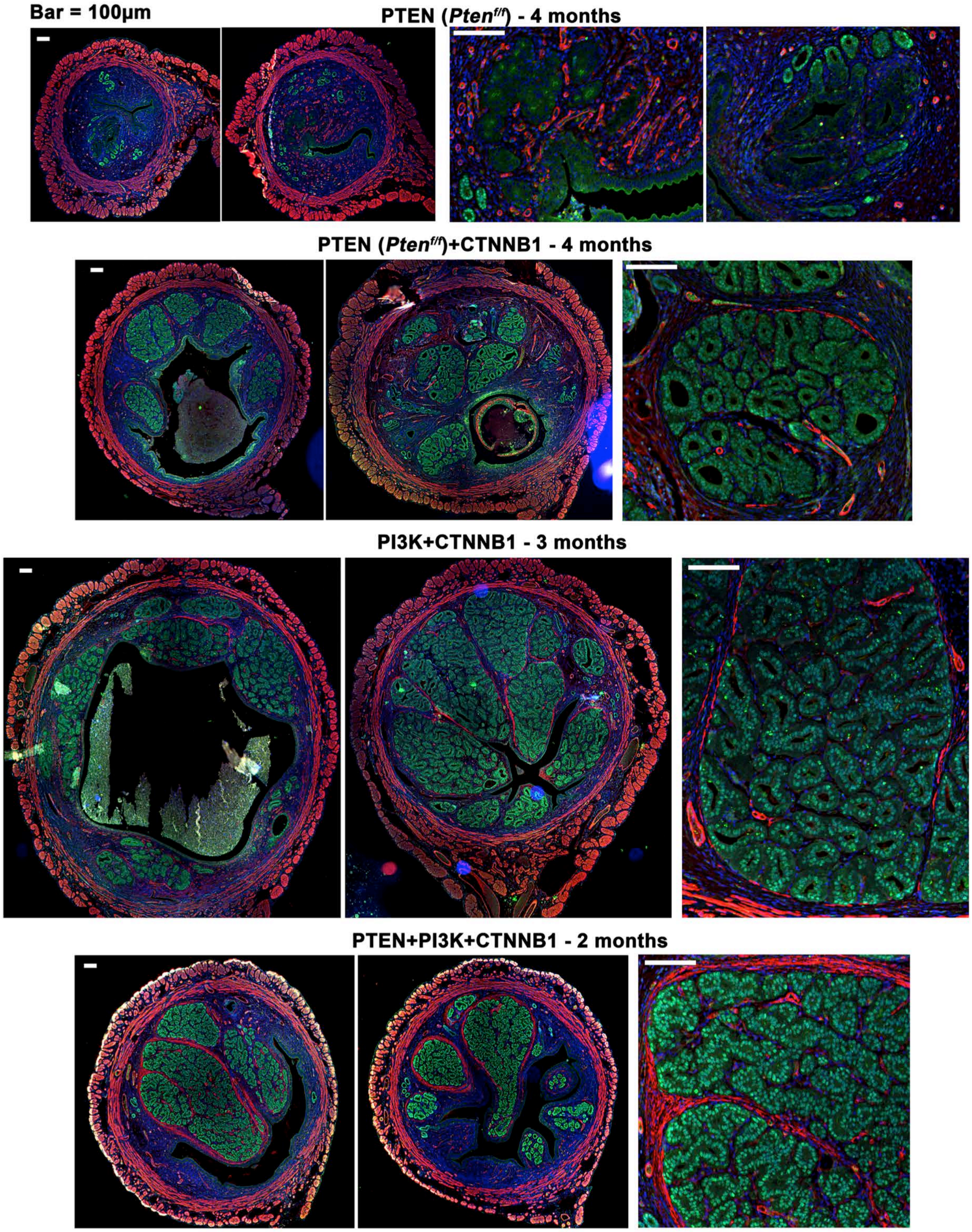

Fig. S6. Distribution of smooth muscle cells/myofibroblasts in the uterine epithelial lesions of single-gene (*Pten*$^{f/f}$) mutant and double-gene/triple-gene mutant mice carrying the *Ctnnb1* mutant allele.

ACTA2 (red) and FOXA2 (green) highlight smooth muscle cells/myofibroblasts and glandular epithelial cells, respectively. The ACTA2-positive layers associated with glandular hyperplasia lesions are particularly prominent in triple-gene mutant mice.

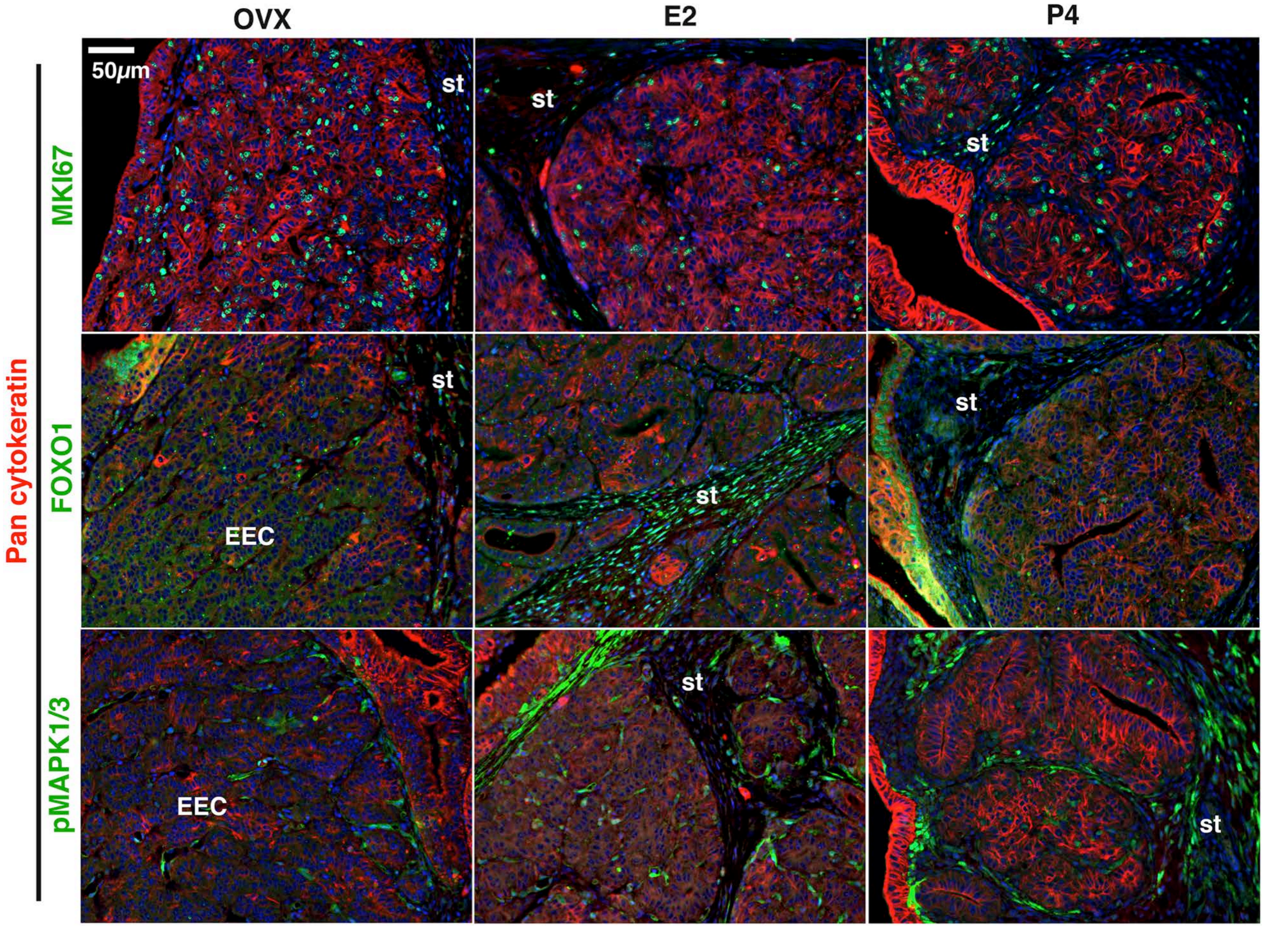

Fig. S7. Effects of ovarian hormones on triple-gene mutant lesions.

st, stroma. EECs in the OVX group lost the most stromal compartment. In the hyperplastic lesions of OVX+E2 and OVX+P4 groups, proliferation (MKI67 expression) was detected in both stromal and epithelial cells. E2 induced nuclear FOXO1 expression in the stromal cells associated with hyperplasia whereas P4 induced FOXO1 in normal UtE. In all groups, pMAPK1/3 was enriched in endothelial cells. In the OVX+E2 and OVX+P4 groups, pMAPK1/3 was also elevated in sub-epithelial stromal cells.

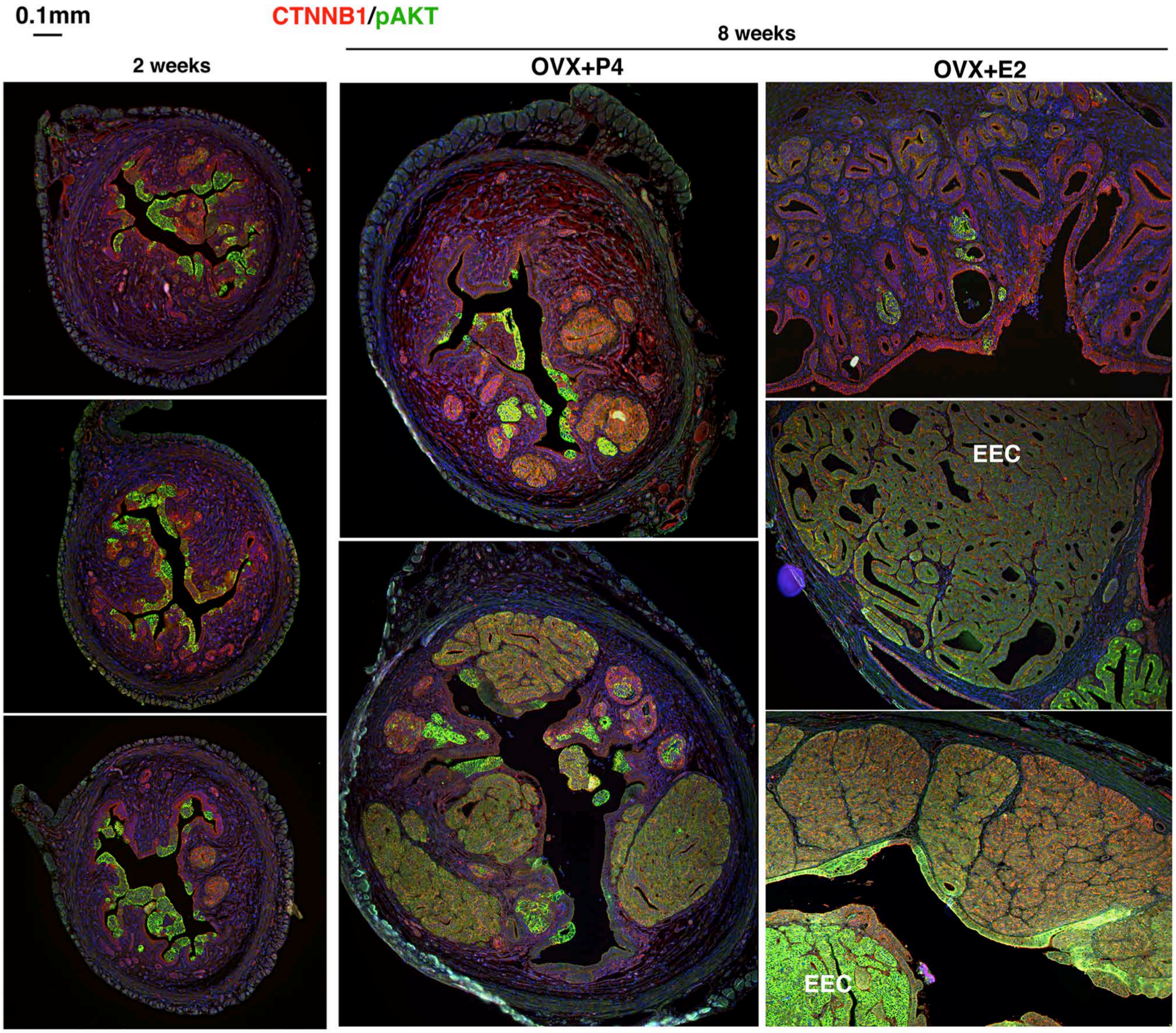

Fig. S8. Progression of epithelial lesions from 2 weeks to 8 weeks after mutagenesis in triple-gene mutant mice of OVX+P4 and OVX+E2 groups.

The time point of 2 weeks represents before castration. Two weeks after injection of 10^8 PFU/ml Ad-Cre, triple-gene mutant mice were castrated and supplemented with P4 or E2 pellet (45), and the epithelial lesions were analyzed 6 weeks later. Mutant cells were highlighted with pAKT (green). Uterine epithelial lesions in these two groups were significantly smaller compared to the OVX group. However, hyperplastic glands enlarged from 2 weeks, and EECs developed in some mice from the OVX+E2 group.

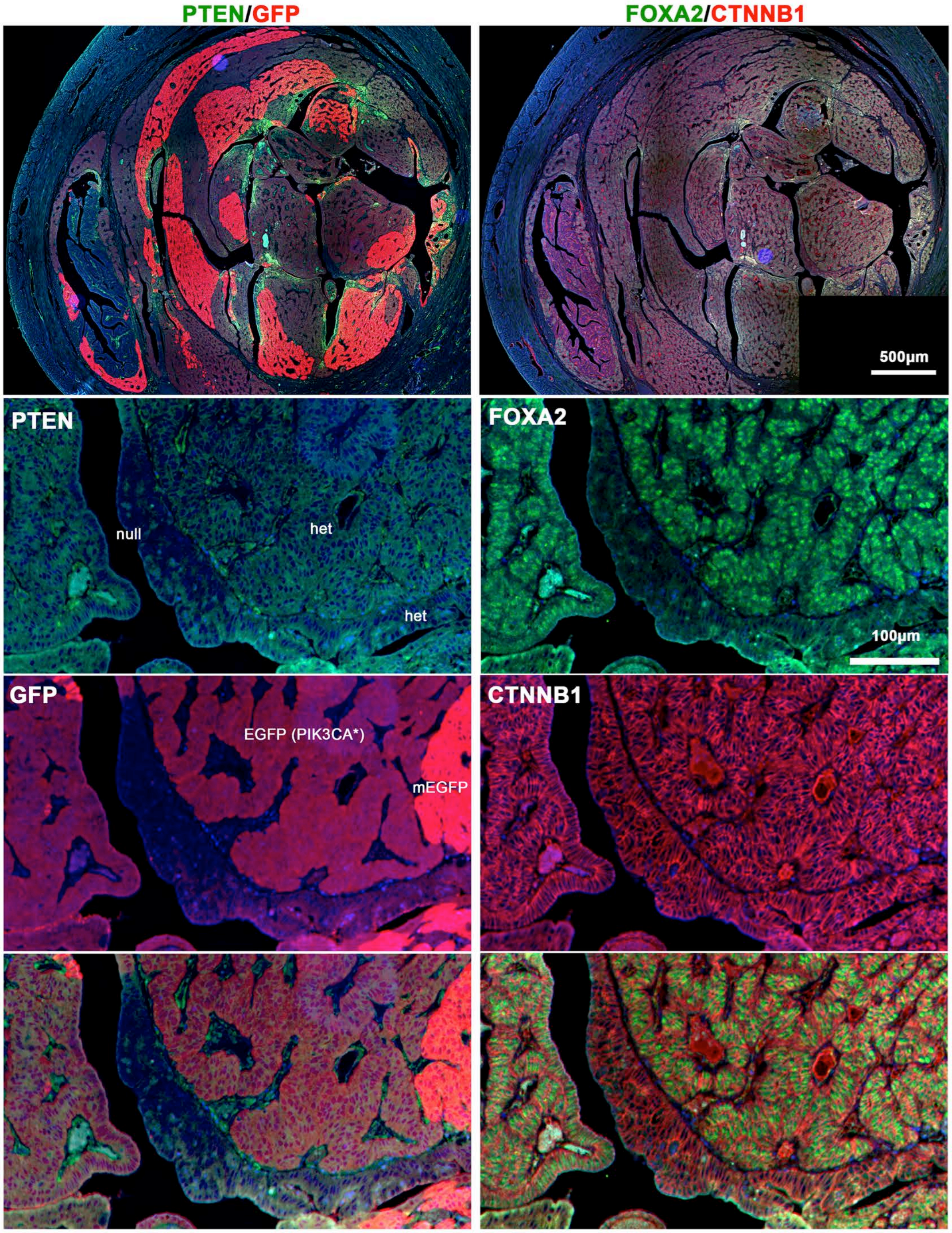

Fig. S9. Epithelial heterogeneity in the uterus of ovariectomized triple-gene mutant mice at 8 weeks after Ad-Cre injection.

There were clusters of epithelial cells lacking PTEN (*), mEGFP, PIK3CA* (EGFP) and/or FOXA2 within the uteri of triple-gene mutant mice. Panels at high magnification show a patch of PTEN null, PIK3CA*-negative and FOXA2-negative cells.
